# HX-Linear and Nonlinear Optical Responsiveness of Rationally Designed Heteroleptic d^8^-Metallo-dithiolene Complexes

**DOI:** 10.3390/molecules30194004

**Published:** 2025-10-07

**Authors:** Salahuddin S. Attar, Flavia Artizzu, Luca Pilia, Angela Serpe, Alessia Colombo, Claudia Dragonetti, Francesco Fagnani, Dominique Roberto, Daniele Marinotto, Paola Deplano

**Affiliations:** 1 Dipartimento di Scienze Chimiche e Geologiche, Università di Cagliari, I-09042 Monserrato, Italy; 2 Department of Chemical Engineering, Texas A&M University at Qatar, Doha 23874, Qatar; 3 Department of Sustainable Development and Ecological Transition (DISSTE), University of Eastern Piedmont, I-13100 Vercelli, Italy; 4 Dipartimento di Ingegneria Meccanica, Chimica e dei Materiali, Università di Cagliari, Via Marengo 2, I-09123 Cagliari, Italy; 5 Dipartimento di Ingegneria Civile, Ambientale e Architettura, Università di Cagliari, Via Marengo 2, I-09123 Cagliari, Italy; 6 Dipartimento di Chimica, Università degli Studi di Milano e UdR-INSTM di Milano, Via C. Golgi 19, I-20133 Milan, Italy; 7 CIMaINa, Università degli Studi di Milano, Via G. Celoria 16, I-20133 Milan, Italy; 8 Istituto di Scienze e Tecnologie Chimiche “Giulio Natta” (SCITEC), Consiglio Nazionale delle Ricerche (CNR), Via C. Golgi 19, I-20133 Milan, Italy

**Keywords:** metal–dithiolene complexes, vaphochromism, nonlinear optics, HX chromic sensors, NLO switch, second harmonic generation

## Abstract

This work presents the HX-responsiveness of the following heteroleptic donor–M–acceptor dithiolene complexes: Bu4N[MII(L1)(L2)] [M = Ni(**1**), Pd(**2**), Pt(**3**)], where L1 is the chiral acceptor ligand [(R)-α-MBAdto = chiral (R)-(+)α-methylbenzyldithio-oxamidate] and L2 is the donor ligand (tdas = 1,2,5-thiadiazole-3,4-dithiolato). Addition of hydrohalic acids induces a strong bathochromic shift and visible color change, which is fully reversed by ammonia (NH_3_). Moreover, the sensing capability of **1** was further evaluated by deposition on a cellulose substrate. Exposure to HCl vapors induces an evident color change from purple to green, whereas successive exposure to NH_3_ vapors fully restores the purple color. Remarkably, cellulose films of **1** were revealed to be excellent optical sensors against the response to triethylamine, which is a toxic volatile amine. Moreover, the HCl-responsiveness of the nonlinear optical properties of complexes **1**, **2**, and **3** embedded into a poly(methyl methacrylate) poled matrix was demonstrated. Reversible chemical second harmonic generation (SHG) switching is achieved by exposing the poled films to HCl vapors and then to NH_3_ vapors. The SHG response ratio HCl–adduct/complex is significant (around 1.5). Remarkably, the coefficients of the susceptibility tensor for the HCl–adduct films are always larger than those of the respective free-complex films. Density Functional Theory (DFT) and time-dependent DFT calculations help in highlighting the structure–properties relationship.

## 1. Introduction

Sensors based on receptors that maintain reversibility and stability of color change when interacting with suitable substances, including halogenides and/or amines, are objects of interest in the framework of environmental monitoring applications [1,2,3,4,5] Among these, several square-planar Pt(II) complexes have been investigated for their potential applications for the detection of water, methanol, dichloromethane, acetonitrile, and nitrate ions [6,7,8,9,10]. A variety of anion receptors have been thoroughly investigated, including those containing hydrogen-bond-donor (HBD) groups. Among these, thiourea-based molecules are suitable for binding anions such as halogenides, whose removal is of interest from industrial, biological, and environmental perspectives. In these cases, anions are linked to the receptor through hydrogen bond interactions [11]. Secondary dithiooxamides, despite the presence of potential HBD groups, exhibit steric unfavorable features to work as halogenide receptors. The steric unfavorable features can be converted into favorable ones when these ligands are coordinated to a platinum(II) ion, which works as a templating agent to provide NH binding sites suitable for effective anion interaction [12]. A variety of relevant properties of the above cited complexes have been pointed out by Lanza, Campagna, and Giannetto [13,14,15,16]. It was established that their behavior is relatable to the steric requisites of secondary dithiooxamides, where the NH groups of the two coplanar thioamide moieties are arranged opposite to each other, while the chiral torsion induced in the secondary dithiooxamide by the *S,S*-coordination to platinum allows the NH groups in a suitable reciprocal disposition to coordinate a chloride anion in a N–H···Cl interaction in the form of tight-ion pair adducts.

**Figure 1:**
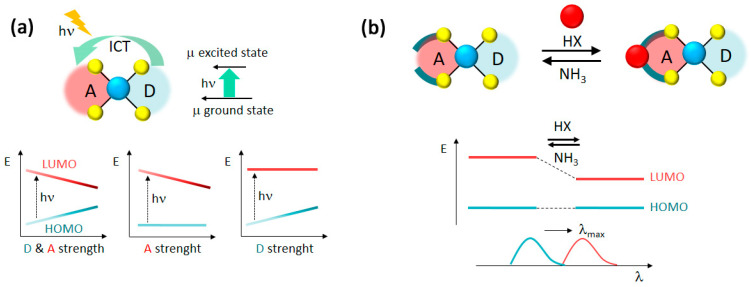
(**a**) Scheme of the optical behavior of heteroleptic square-planar d^8^-metal dithiolene complexes bearing a donor (D) and an acceptor (A) ligand (above), and diagrams of the HOMO (blue)–LUMO (red) gap on variation in the D/A strength. (**b**) Optical chromism of D/A d^8^-metal dithiolene systems, where A is responsive to hydrohalic acids (HX), with representative energy level diagrams of frontier MOs.

These tight ion-paired adducts can donate a HCl molecule to a suitable base such as an amine, restoring the early complex. Since the reagent and the product show different absorptions in the visible region, these tight ion-paired adducts have been successfully employed as self-indicating titrants in the spectrophotometric determination of aliphatic amines of pharmaceutical interest, which are known to be visibly transparent in the visible spectrum. Remarkably, the capacity to exhibit a visual color change when exposed to pH variation stimuli makes these molecules suitable candidates as HX-chromic sensors [15,16]. The templating properties towards dithiooxamides of platinum(II), extended to other d^8^-metal ions, can be further exploited by employing these ligands as component of different mixed-ligand complexes, such as d^8^-metal–heteroleptic dithiolene complexes. As is well known, the term dithiolene is employed irrespective of the real form of the ligand, which can bind through a chelating C_2_S_2_ group a variety of metals such as neutral dithioketone, mixed-valence thioketone-thiolate monoanion, and ene-1,2-dithiolate dianion, depending on the nature of the substituents at the C_2_S_2_ moieties. In fact, the substituents affect the energy of the frontier orbitals, pushed up or down by electron-donating or electron-withdrawing groups, respectively [17,18,19,20]. By proper selection of the substituents, heteroleptic dithiolenes can be thus described even as dithione–dithiolate derivatives. This versatile class of chromophores containing a π-donor (D, a dithiolato, contributing mainly to HOMOs) and a π-acceptor (A, dithiooxamides or dithiooxamidate, contributing mainly to LUMOs) ligand connected by a d^8^-metal in a square-planar coordination is characterized by a donor–acceptor (D-A) Intramolecular Charge-Transfer (ICT) transition, which gives rise to intense absorption in the visible spectral region. The metal acts as a suitable π-bridge for the D-A ICT-transition, with a decrease in the dipole moment from the ground to the excited state, as shown in Figure 1a, reflected by a negative solvatochromism [17,18,19,20].

These chromophores show linear optical properties that are tunable with the rational design of ligands. Thus, as sketched in Figure 1a, the energy of the ICT transition, and consequently, the wavelength of the related peak, is tunable with the donor and acceptor strength. This can be achieved by proper selection of both component ligands or by keeping the acceptor constant on variation in the donor, or inversely, as summarized in Figure 1a. In addition, when a functional group, such as an amidate moiety, is integrated in the periphery of the ligands (Figure 1b) and is capable of responding to external stimuli, the complex shows, in addition to solvatochromism, additional responsiveness to HX, becoming a multiresponsive chromophore.

Accordingly, in these chromophores, where a dithiooxamidate ligand provides in the periphery of the acceptor the site to interact with hydrohalic acids, tight ion-paired adducts are formed (Figure 1b). In agreement with expectations, previous results on Bu_4_N[M((*R*)-α-MBAdt)(L)] (M = Pd^II^, Pt^II^; MBAdt = methylbenzyldithiooxamidate; L = dmit (2-thioxo-1,3-dithiole-4,5-dithiolato), ddmet = 1,2-dicarbomethoxyethylene dithiolate, quinoxdt = [4′,5′:5,6][1,4]dithiino [2,3-b]quinoxaline-1′,3′dithiolato) have shown that these complexes behave as HCl-switchable chromophores characterized by a shift to a lower energy of the peak relatable to the D-A ICT transition [21].

Remarkably, in solution, these d^8^-metal–heteroleptic dithiolene complexes are also characterized by a good second-order nonlinear optical (NLO) response that increases significantly upon HCl addition, in agreement with a decrease in the HOMO-LUMO gap [21]. This observation is relevant in the field of nonlinear optics [22] because introduction of switchability into the NLO behavior of compounds increases their potential for novel applications in emerging optical and sensing technologies [23,24,25,26,27,28,29,30]. In addition, it is worth pointing out that although the great potential of metal complexes as multifunctional molecular materials has been confirmed by many studies in solution [31,32,33,34,35,36], their nano-organization in polymeric films with the aim of obtaining composite films with switchable linear and nonlinear optical properties is still in its infancy and needs to be developed in view of photonic applications.

These observations encouraged us to design new donor ligands to extend the color palette of the acido-chromic response of palladium and platinum heteroleptic dithiolene complexes and to investigate the nano-organization of these kinds of NLO-phores in polymeric matrixes to achieve films characterized by a reversible HCl-/NH_3_-switchable second-harmonic generation.

Moreover, the high earth abundance and lower cost production of nickel derivatives, in comparison with the corresponding Pd- and Pt-ones, prompted us to include the Ni metal in these studies. We found that the use of Bu_4_N[M((*R*)-α-MBAdt)(tdas)] [M = Ni (**1**), Pd (**2**), Pt (**3**), tdas =1,2,5-thiadiazole-3,4-dithiolato] allows for the achievement of a color palette extension as HX sensors in agreement with a rational tuning of colors in the function of the donor component, whose role in the bonding has been deepened by Density Functional Theory (DFT) calculations. The (i) successful preparation of a novel Ni-complex, (ii) processing of the pure complexes as films on paper or dispersed in poly(methyl methacrylate) (PMMA) on glass substrates able to detect volatile hydrohalic acids and organic amines with naked-eye color changes, and (iii) achievement of reversible HCl-/NH_3_-switchable second-harmonic generation of the complexes oriented in PMMA polymeric films are relevant in providing versatile candidates for smart optical devices and chemo-sensors.

## 2. Results and Discussion

### 2.1. Preparation of Complexes

**Scheme 1:**
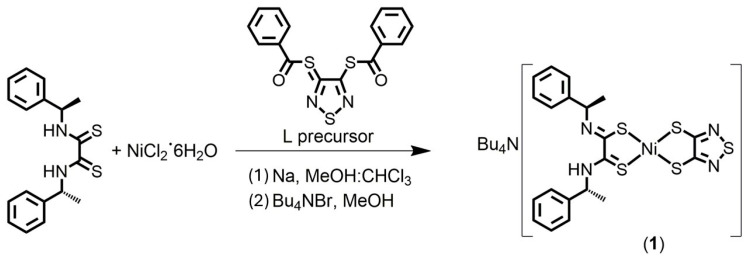
Preparation of the investigated Ni complex.

**Scheme 2:**
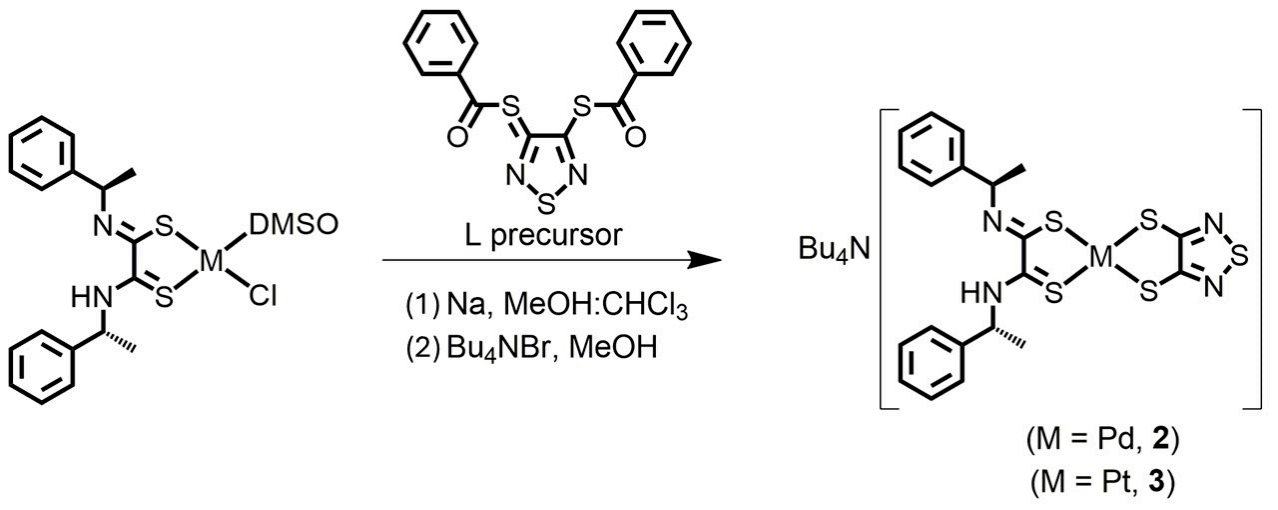
Preparation of the investigated Pd and Pt complexes.

The preparation of the Bu_4_N[M((*R*)-α-MBAdt)(tdas)] complexes is summarized in Scheme 1 for M = Ni (**1**) and in Scheme 2 for M = Pd (**2**) and M = Pt (**3**).

In the nickel case (Scheme 1), the (*R*)-α-MBAdto ligand and the Ni-salt, in a 1:1 molar ratio, were mixed in methanol and stirred for 30 min; after that, the tdas ligand was obtained by deprotection of the ligand precursor by sodium methoxide in a MeOH/CHCl_3_ solvent mixture. On treatment of the reaction mixtures with tetrabutyl ammonium bromide and purification by crystallization in acetonitrile/diethyl ether or ethanol, Bu_4_N[Ni((*R*)-α-MBAdt)(tdas)] was obtained in a 53% yield. The above one-pot reaction does not work with the corresponding Pd/Pt halogenides, and the Pd/Pt desired mixed ligand complexes are obtained by employing reactive intermediate Pd/Pt-complexes, as shown in Scheme 2. These intermediates, prepared in chloroform solutions in a first step, are employed to react with the tdas ligand in a MeOH/CHCl_3_ solvent mixture at room temperature in a 1:1 molar ratio. On treatment of the reaction mixtures with tetrabutyl ammonium bromide and purification by crystallization in acetonitrile/diethyl ether or ethanol, the tetrabutyl ammonium salts of the complexes were obtained in 54% (Pd) and 41% (Pt) yields.

### 2.2. Linear Optical Properties and DFT Calculations

**Figure 2:**
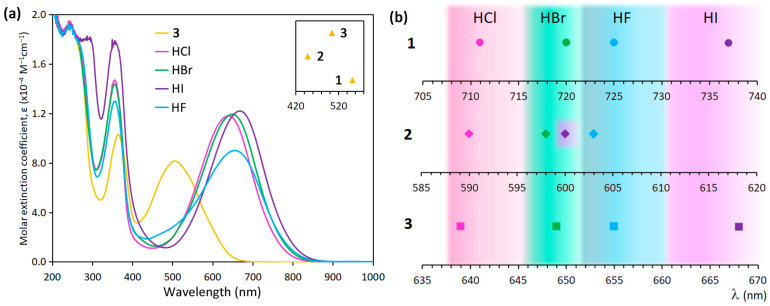
(**a**) Electronic absorption spectra of Bu_4_N[Pt((*R*)-α-MBAdt)(tdas)] (**3**) in ACN (yellow, conc. = 2.5·10^−4^ M) and of its 1:1 HX adducts (HCl = pink; HBr = green; HF = blue; HI = purple). The inset reports a comparison of the absorption maxima of the lowest absorption band in the visible range of complexes **1**–**3**. (**b**) Positions of the absorption maxima of the visible band in complexes **1**–**3** upon addition of the different hydrohalic acids.

The electronic absorption spectra of **3** in acetonitrile (ACN) solution, as shown in Figure 2a, are characterized in the visible region by two main bands: one broad medium-intensity peak at 505 nm with a more or less resolved shoulder at higher frequency and a quite intense absorption band below 400 nm. Upon addition of hydrohalic acids, the color of the solution changes, and the lowest absorption band, relatable to the CT donor–acceptor transition, is shifted to higher wavelengths for a 1:1 molar ratio between HX and the complex. Similar behavior is observed for **1** and **2**, and the spectral changes for HX addition to their ACN solutions are reported in Figure 2b.

**Figure 3:**
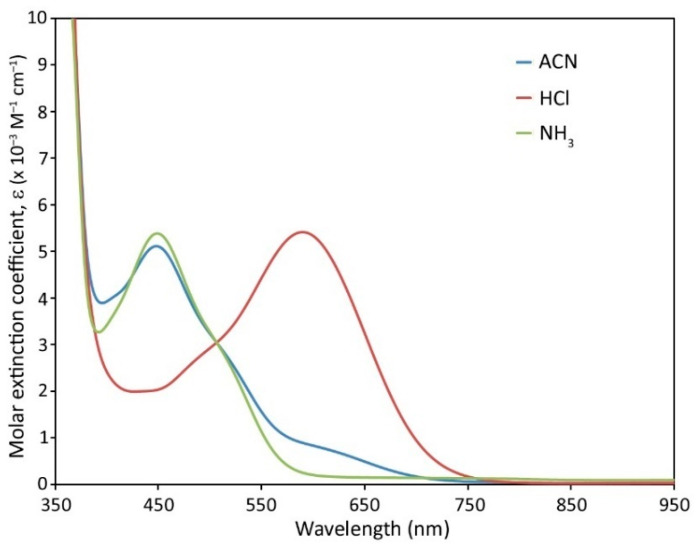
Reversibility of the electronic absorption of Bu_4_N[Pd((*R*)-α-MBAdt)(tdas)] (**2**) in ACN (blue, conc. =2.5·10^−4^ M) upon the sequential addition of an equimolar amount of HCl (red) and NH_3_ (blue).

The transformation is reversible, and upon NH_3_ addition, when a 1:1 molar ratio with respect to the HX is reached, the original spectra are recovered, as shown in Figure 3 for **2** as an example. The corresponding spectra for **1** and **3** are reported in Figure S5. It is worth noting that, in the case of **1**, the transformation is not fully reversible in solution. However, as further discussed in Section 2.3, full reversibility is achieved, as the Ni-complex is stabilized through deposition on a cellulose substrate.

**Figure 4:**
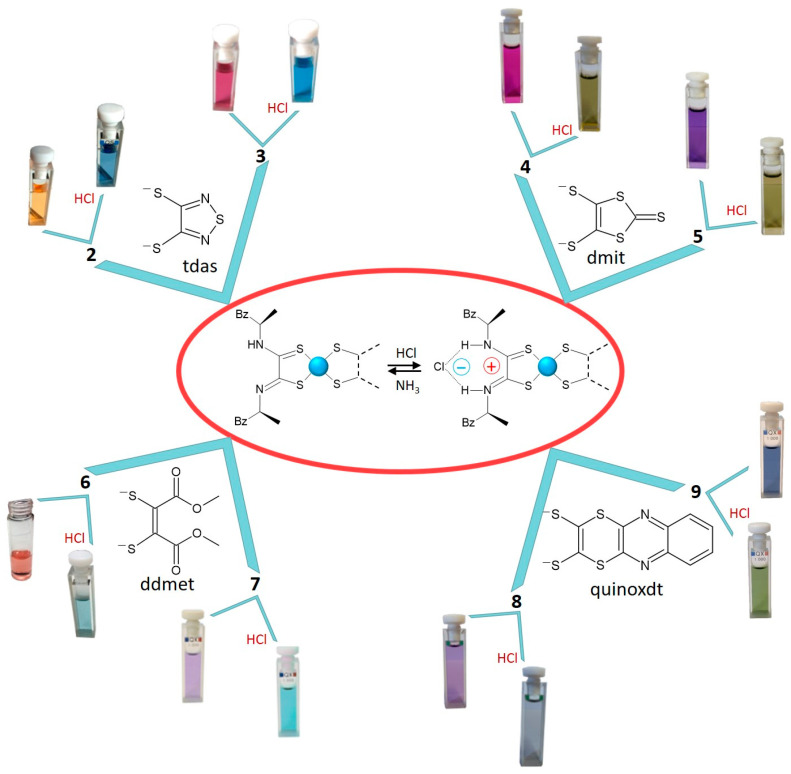
Color tunability of Bu_4_N[M((*R*)-α-MBAdt)(L)] derivatives in ACN solution on variation in the sonor L ligand (structures reported in the insets) and upon addition of equimolar amounts of HCl. The mechanism of the formation of the hydrochloride adduct through a tight contact ion pair of the acceptor (*R*)-α-MBAdt ligand is also reported in the center.

Figure 4 reports a summary of the color variation upon HCl addition in Bu_4_N[M((*R*)-α-MBAdt)(L)] derivatives, where M = Pd, Pt and L = tdas, **2**, **3**; dmit **4**, **5**; ddmet **6**, **7**; quinoxdt **8**, **9**, whereas visible peak wavelengths and the corresponding literature references are listed in Table 1.

The electronic structures of the anionic complexes **4**–**9** have been previously elucidated by means of DFT methods [21]. As anticipated in the Introduction, in all complexes, the HOMO is mainly localized on the dithiolate system, whereas the LUMO is mainly localized on the dithio-oxamidate fragment. According to time-dependent DFT calculations, the low-energy absorption band can be associated almost exclusively to a HOMO → LUMO transition. The most favorable protonation site is the nitrogen atom of the dithiooxamidato ligand forming a N-H···Cl hydrogen bond with a more symmetrical shape of the Mos, which stabilizes the LUMO, resulting in a decrease in the HOMO-LUMO energy gap. Thus, the presence of one equivalent of HCl per complex molecule promotes a red shift in the low-energy absorption band, as found.

With the acceptor being common in the complexes under discussion, the sequence of the absorbances of the ICT peak, relatable to the HOMO-LUMO transition, is mostly relatable to the donor properties.

Table 1 shows that, by employing the tdas donor, related complexes exhibit a shift towards lower wavelengths when compared to the corresponding ones with the other donor ligands so far investigated, suggesting lower donating properties for this ligand.

To provide support to the experimental findings, these complexes were investigated by means of DFT methods, both in the gas phase and CH_3_CN. The computational studies were performed using the functional Becke three-parameter exchange and Lee–Yang–Parr correlation (B3LYP) [37,38] along with the basis set 6-31+G** [39] for H, C, N, and S atoms and the LANL2DZ [40] (Ni) and Def2TZVPP [41,42] (Pd and Pt) ECP basis sets for the metal ions. For all the investigated complexes, the optimized geometries show molecules with the metal ion, tdas, and the N_2_C_2_S_2_ moiety of the (*R*)-α-MBAdt ligand lying in the same plane (Figure S6). As far as the molecular orbitals (MOs) are considered (Tables S1–S3), it can be observed that the HOMOs are antibonding *π*-orbitals, mainly formed by the tdas ligand with contributions from the dithiooxamidate and from the metal. The LUMOs are also *π*-orbitals, but they are composed of the (*R*)-α-MBAdt ligand with an almost negligible contribution from the metal. TD-DFT calculations were performed to gain further insight into the electronic transitions (Figures S7–S9 and Tables S4–S6). The predicted absorption maxima are in satisfactory agreement with the experimental findings. For complex **1**, the absorption observed at the longest wavelength (563 nm) can be attributed to three calculated transitions at 491, 486, and 432 nm, with oscillator strengths (*f*) of 0.0722, 0.0932, and 0.0108, respectively. In the case of complexes **2** and **3**, the experimental absorption bands at 448 nm (**2**) and 505 nm (**3**) are reproduced by two transitions: for 2, at 499 nm (*f* = 0.1550) and 441 nm (*f* = 0.0166); and for **3**, at 541 nm (*f* = 0.152) and 477 nm (*f* = 0.1095). Moreover, as previously found for complexes **4**–**9**, TD-DFT calculations predicted that the absorptions at lower energies shown by these complexes are mainly due to HOMO-LUMO transitions (Figures S7–S9 and Tables S4–S6), pointing to a donor–acceptor character of these electronic transitions. It is worth noting that this electronic feature plays an important role in the second-harmonic generation [21].

**Figure 5:**
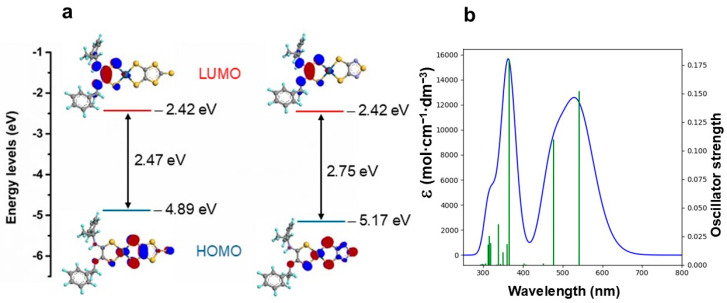
Comparison of frontier orbital energies in dmit (left) and tdas (right) corresponding derivatives in the case of platinum complexes (**a**). TD-DFT-simulated absorption spectrum for compound **3** in acetonitrile solution (**b**).

With the aim of evaluating the influence of the electrondonating properties of the tdas ligand, a comparison with the corresponding complex bearing the dmit as donor ligand was carried out. Indeed, the complexes of this ligand seemed to us to be the most suitable among those reported in Table 1, since, in both ligands, the substituents at the dithiolene core maintain similar structural features consisting of a condensed pentatomic ring. In Figure 5, the calculated frontier orbitals of complex **3** are reported and compared with those of the corresponding [Pt((*R*)-α-MBAdt)(dmit)]^−1^ (**5**). As expected, since the acceptor ligand is the same in both complexes, the LUMOs are identical in shape and energy. The HOMOs instead present energy values of −5.17 and −4.89 eV for tdas and dmit, respectively. Consequently, the dmit complex presents a smaller HOMO-LUMO gap (2.75 vs. 2.47 eV), in agreement with the λ_max_ shift to lower wavelengths observed for the tdas complex by comparison of the UV–vis spectra (Table 1), suggesting a lower donating capability of the tdas ligand. Clearly, a computational approach can provide important support for a rational donor ligand design to tailor the chromophore band gap in order to tune absorption across the visible spectrum, allowing for the creation of desired color tunable candidates for molecular sensing.

### 2.3. Linear Optical Sensing

**Figure 6:**
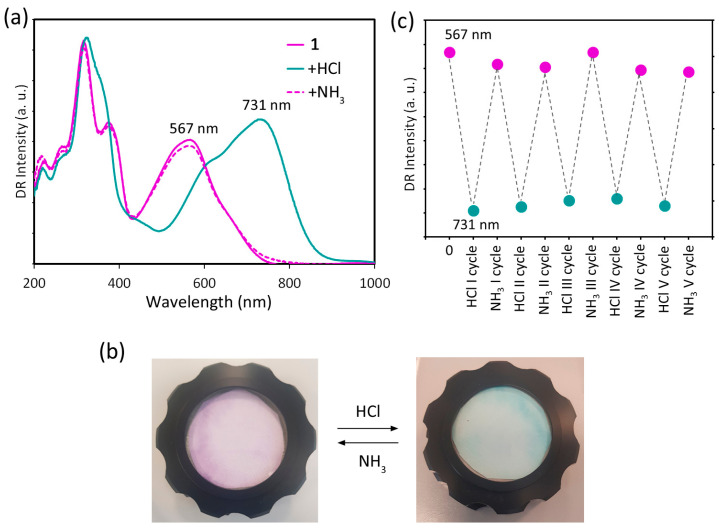
(**a**) Diffuse reflectance spectra (reported in absorbance units) of **1** deposited on a cellulose support (solid purple line) and subsequently exposed to vapors of HCl (green line) and NH_3_ (purple dotted line). (**b**) Photographs of the paper sensor before (left) and after (right) exposure to HCl vapors. Further exposure to NH_3_ vapors restores the initial purple color. (**c**) Relative variation in the absorption intensity of the lowest energy band of **1** deposited on a cellulose support over 5 cycles of HCl/NH_3_ vapor exposure. The small fluctuations in the spectral intensity are mainly attributed to variations in the positioning of the sample with respect to the integrating sphere in the diffuse reflectance experiments.

In the case under discussion (D = tdas), we selected the nickel derivative (**1**) for its attractiveness given Ni’s abundance and low cost with respect to corresponding Pd and Pt compounds. This complex was deposited on a cellulose substrate to check its ability to respond to HCl and NH_3_ vapors. A deep-purple complex solution was prepared by dissolving the complex in CH_2_Cl_2_. The solution was drop-casted on filter paper, which was dried in air. Exposure to HCl (37%) vapors induces an evident color change from a purple color to a green color, with full color tuning taking place over approximately 2 min, as shown in Figure 6a,b. Successive exposure to NH_3_ vapors (30%) fully restores the purple color in 10–15 s.

Strikingly, the sensing system showed full reversibility after five cycles of HCl/NH_3_ exposure, with no changes in color of the original compound and its hydrochloride adduct, as also confirmed by diffuse reflectance spectral data shown in Figure 6c (full spectra are reported in Figure S10).

It is worth mentioning that the corresponding film obtained by solution drop-casting of the pure complex on a glass support showed the same results observed on paper in terms of color change and reversibility (Figure S11) but with significantly faster response times (<10 s for the response to HCl). However, owing to the tendency of the complex to form aggregates, the film deposited on glass is poorly homogeneous. On the other hand, a cellulose support, despite yielding slower response times, represents a flexible, cheap and convenient alternative for sensor integration in applicative purposes, such as, for example, product packaging. In this context, the optical responsiveness of **1** casted on paper in its hydrochloride adduct form was also successfully tested against the response to triethylamine (Figure S12), which is a toxic volatile amine widely used in agriculture, the textile industry, etc., and is an indicator for seafood freshness [43]. Based on the evident response, which is also fully reversible upon exposure to HCl vapors, **1** deposited on a cellulose support can be proposed as an efficient and economic sensing system for food quality, which can be easily incorporated in food packaging.

The spectrophotometric results also further demonstrate that the vapochromic behavior of the films is in agreement with the formation of stable, stoichiometric adducts.

In conclusion, these multicolor HX–receptor complexes can work as sensors for the naked-eye detection of hydrohalic acids or, in the HX–adduct form, of visible transparent amines both in solution and on paper substrates.

### 2.4. Nonlinear Optical Properties

As shortly summarized in the introduction, properly designed square-planar heteroleptic metal dithiolene complexes show a large quadratic hyperpolarizability, as determined by the Electric-Field-Induced Second-Harmonic generation (EFISH) technique [17,18,19,20,21,44]. Remarkably, when an amidate moiety is integrated in the periphery of the ligands (Figure 1b), the complex shows responsiveness to HX [21]. A reversible chemically switchable second-order NLO response is determined in solution, with an increase in the quadratic hyperpolarizability upon HCl addition due to a decrease in the HOMO-LUMO gap caused by a shift to lower energy of the LUMO (Figure 1b), whereas addition of NH_3_ restores the original *β* value [21]. Attempts to obtain films characterized by a reversible chemically switchable NLO response failed [21]. Remarkably, the Bu_4_N[M((R)-α-MBAdt)(tdas)] [M = Ni (**1**), Pd (**2**), Pt (**3**)] complexes prepared in the present work represent the first examples of metal dithiolene complexes suitable for providing composite films with a chemically switchable stable and large second-harmonic generation.

**Figure 7:**
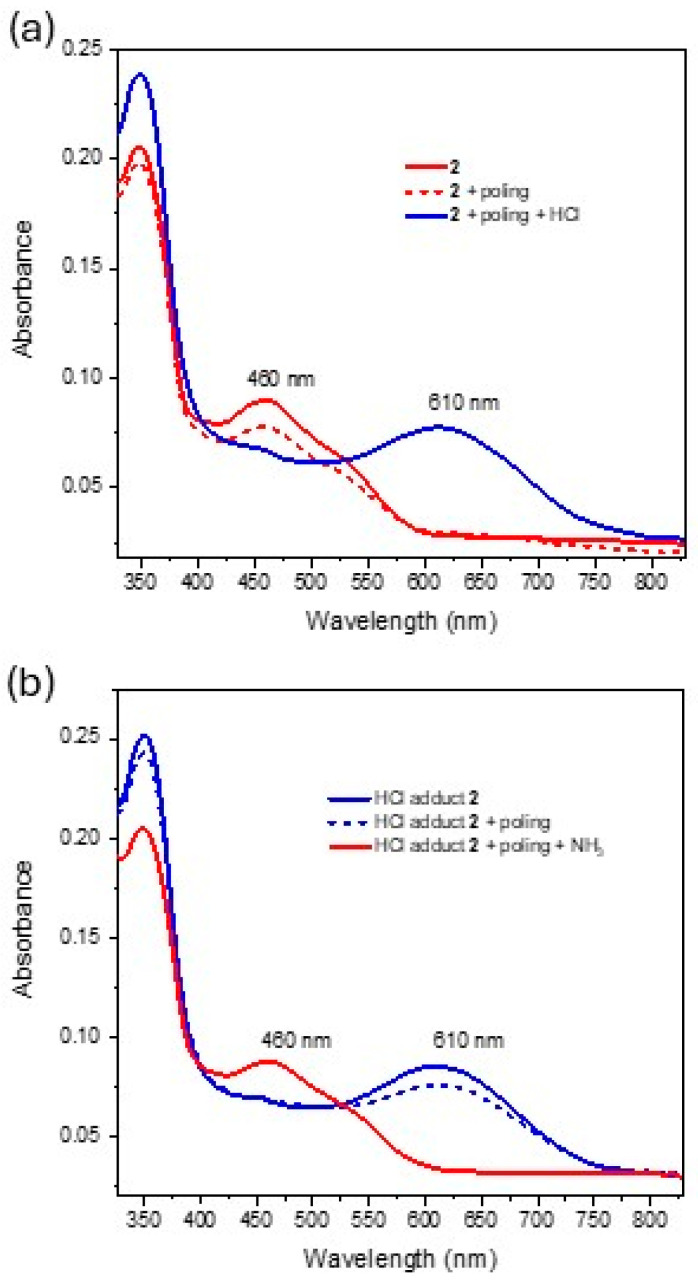
UV−vis absorption spectra of Pd (**2**) films (**a**) and related HCl–adduct films (**b**) before and after poling with relative switch.

Composite thin films of complexes **1**–**3** in a PMMA matrix were prepared as reported in the Materials and Methods section. The UV−vis absorption spectra of these films before poling are characterized by a broad band in the visible region at 566, 460, and 520 nm for the Ni, Pd, and Pt films, respectively (for the Pd film, Figure 7a; for the Ni and Pt films, see Figure S13). These absorption bands are related to the charge transfer transition from the donor to the acceptor ligand, as already observed (Section 3.2) for the complexes in DMF solutions. They are shifted to higher wavelengths (broad band at 730, 610, and 659 nm for the Ni, Pd, and Pt films, respectively) upon exposure of the films to HCl vapors (37%), in agreement with the formation of the related HCl–adduct complexes (for the Pd film, see Figure 7b; for the Ni and Pt films, see Figure S13).

**Figure 8:**
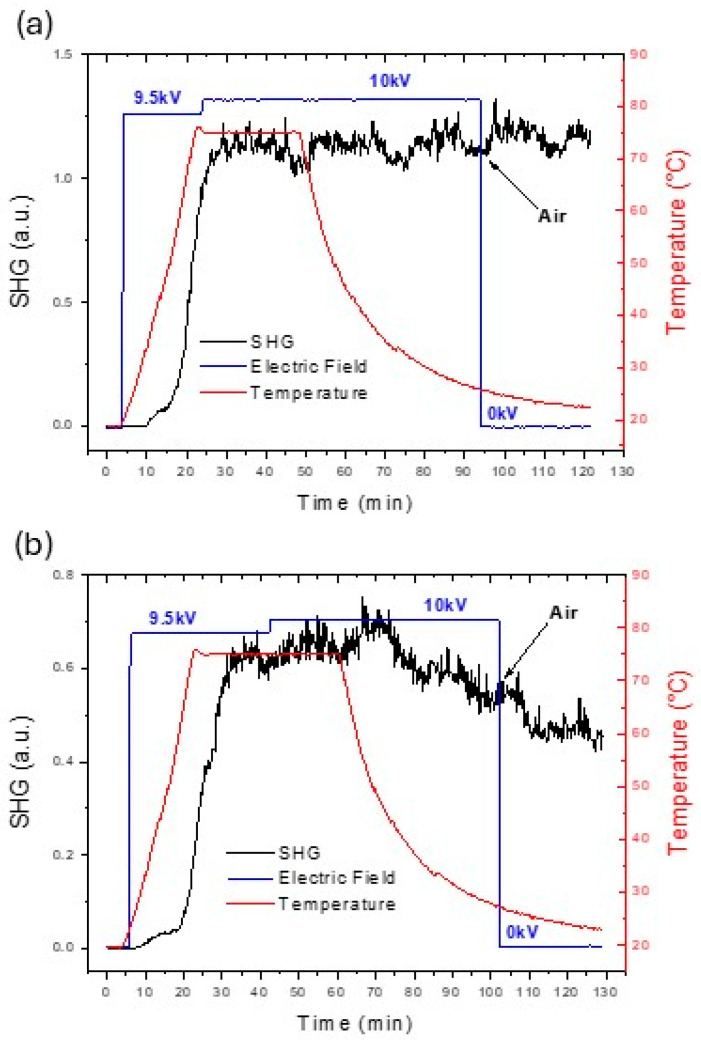
Corona-wire poling dynamics of (**a**) **2** film and (**b**) HCl–adduct **2** film.

The second-order NLO properties of the films based on complexes **1**, **2**, and **3** and those of the related HCl–adduct films were induced by applying the corona-wire poling technique (see the Materials and Methods section), which was necessary to orient the dipolar molecules in the PMMA matrix. In order to avoid degradation of the film and to increase the SHG signal as much as possible during the poling, it was necessary to optimize the temperature and the applied electric field. The best corona-wire poling dynamics conditions of **1**, **2**, **3**, and the related HCl–adduct films are shown in Figure 8 (for Pd films) and Figure S14 (for Ni and Pt films). All analyzed films display a quite general poling behavior: the SHG signal is negligible at room temperature and increases as the temperature is increased close to the glass-transition temperature (T_g_) of PMMA (86.5 °C) with an applied electric field of 9.5 ÷ 10 kV, reaching its maximum value in about 30–40 min for Ni and Pd films and 110 min for Pt films. This variation in the SHG signal is due to the decrease in the viscosity of the PMMA matrix near the T_g_, which allows for an easier orientation of the chromophores. After the SHG signal reached the plateau, the temperature was decreased to room temperature in order to “freeze” the orientation of the chromophores inside the polymeric matrix; subsequently, the drybox was opened and the electric field was turned off. The absorption spectra of all films were recorded after poling, and they showed the characteristic dichroic effect, in which the broad absorption bands remained unchanged in shape, although they decreased in intensity compared to those recorded before poling (see Figure 7 and Figure S13). This phenomenon is related to the orientation of chromophores along the direction of the electric poling field [45,46]. After poling, no appreciable Stark shift in the absorption peaks was observed [45,46].

By fitting the Maker fringe measurements, using the expressions expected for poled films with C_∞v_ symmetry, the three nonzero coefficients of the second-order susceptibility tensor χ_33_^(2)^, χ_31_^(2)^, and χ_15_^(2)^ were evaluated (Table 2); the error in these data can be estimated at about 20%. From the data in Table 2, we observe that the χ_33_^(2)^ component is always bigger than that of the χ_31_^(2)^ and χ_15_^(2)^ components for all films; this result is generally valid for poled thin films of *push−pull*-type chromophores, in which the charge transfer from the donor to the acceptor ligand is predominantly monodimensional. In addition, the two components χ_31_^(2)^ and χ_15_^(2)^ are not equal to each other within experimental error for each film; therefore, the Kleinman symmetry is not valid. This is due to absorption at 532 nm (second-harmonic wavelength), which is not zero for all investigated films, leading to some resonance enhancement.

Remarkably, the coefficients of the susceptibility tensor for the HCl–adduct films are always larger than those of the respective **1**, **2**, and **3** films, in agreement with a decrease in the HOMO-LUMO gap. The HCl–adduct Pt film displays the highest values: 8.48, 2.48, and 2.02 pm/V for χ_33_^(2)^, χ_31_^(2)^, and χ_15_^(2)^, respectively, which is followed by the HCl–adduct Pd film (4.22, 1.04, and 0.72 pm/V) and the HCl–adduct Ni film (3.20, 0.66, and 0.55 pm/V). The susceptibility tensor for the related **1**, **2**, and **3** films shows the same trend: Pt > Pd > Ni (Table 2). This behavior reflects the quadratic hyperpolarizability trend of these kinds of metal dithiolene complexes in solution, as determined by the EFISH technique, probably due to a better mixing of the orbitals of Pt with those of the dithione and dithiolato ligands and to a larger oscillator strength for the Pt compounds [44].

It is worth pointing out that the χ_33_^(2)^ values of the films based on Bu_4_N[M((*R*)-α-MBAdt)(tdas)] (3.24 and 6.12 pm/V for M = Pd and Pt, respectively) are much higher than those previously reported for PMMA films based on related complexes with a dicarbomethoxyethylenedithiolato (ddmet) instead of 1,2,5-thiadiazole-3,4-dithiolato as a ligand (2.02 and 1.32 pm/V for M = Pd and Pt, respectively) [45,46], giving evidence for the important role of the dithiolato ligand in the SHG response. Actually, it is worth pointing out that the SHG response of the investigated complexes is among the best reported for metal complexes nano-organized in a polymeric matrix [47,48,49,50,51], close to that of Disperse Red One, [*trans*-4,4′-O_2_NC_6_H_4_N=NC_6_H_4_NEt(CH_2_CH_2_OH)], which has been used in electrooptic polymeric poled films [52], often used as a benchmark (χ_33_^(2)^ = 16.7 pm/V).

**Figure 9:**
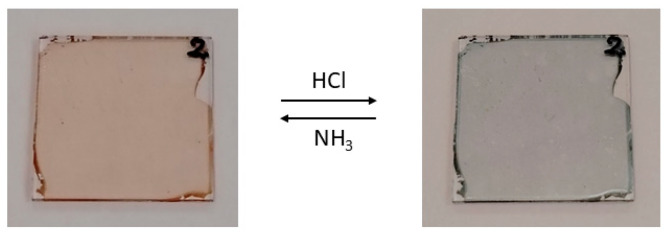
Photographs of **2** in PMMA thin film on glass before (left) and after (right) exposure to HCl vapors. Further exposure to NH_3_ vapors restores the initial red color.

Remarkably, reversible chemical SHG switching is achieved by exposing the poled films based on the Bu_4_N[M((*R*)-α-MBAdt)(tdas)] (M = Ni, Pd, Pt) form to HCl vapors and poled films of the related HCl–adducts to NH_3_ vapors. The SHG response ratio HCl–adduct/free complex is significant, around 1.5 (Table 2). The absorption spectra of these films confirm the transition from the free complex to its HCl–adduct form and vice versa (see Figure 7 and Figure S13), a change that can be also observed by the naked eye (Figure 9).

Moreover, from data in Table 2, we note that the components of the susceptibility tensor after the switching are similar to those obtained directly from poling of the same free complex or HCl–adduct form. Since the measured contrast is sufficiently large to allow for unambiguous distinction between the two forms, these chemical SHG switches based on metal dithiolenes have great potential for applications in the field of photonic and optoelectronic devices. To our knowledge, they represent the first examples of coordination compounds nano-organized and oriented in a PMMA matrix, affording films which can act as reversible HX-/amine-triggered SHG switches.

## 3. Materials and Methods

### 3.1. Synthesis and Characterization of Products

Reagents and solvents of reagent grade and spectroscopic grade were used as received from Sigma-Aldrich. (*R*)-α-MBAdto ligand [53], the tdas precursor [54], and complex [Pt((R)-α-MBAdt)(DMSO)Cl] [21] were prepared according to the literature. A neat reaction between solid rubeanic acid and liquid (*R*)-(+)-α-methyl benzyl amine ((R)-α-MBA) in a 2:1 molar ratio produced the (*R*)-α-MBAdto ligand in a good yield. Purification was carried out by column chromatography on neutral alumina followed by recrystallization in concentrated dichloromethane solutions. Elemental analyses were performed by means of a PerkinElmer CHNS/O PE2400 Series II device (Perkin Elmer, Inc., Shelton, CT, USA). IR spectra (Figure S1) (4000−400 cm^−1^) were recorded with a Jasco FT/IR-6700 Spectrometer (Jasco Corporation, Tokyo, Japan). Electronic spectra were recorded with an Agilent Cary 5000 spectrophotometer. Absorption spectroscopy studies on color variation upon addition of haloacids were performed by adding 50 μL of a 10^−3^ M acetronitrile solution of the haloacid HX to a cuvette containing 2.0 mL of a 2.5·10^−4^ M acetonitrile solution of the corresponding metal complex. Successive additions of 50 μL of 10^−3^ M ammonia solution in acetonitrile recovered the initial spectrum of the metal complex. Spectral intensities were appropriately corrected for the solvent volume variation. ^1^H NMR spectra (Figures S2–S4) were recorded using a Bruker Advance III HD 600 device (Bruker, Bremen, Germany) at 600 MHz using CD_3_CN as a solvent. The chemical shifts (δ) are reported in parts per million downfield from tetramethylsilane.

#### 3.1.1. Bu_4_N[Ni((R)-α-MBAdt)(tdas)] (1)

A solution of NiCl_2_.6H_2_O (100.0 mg, 0.42 mmol) in 25 mL of methanol was added drop-wise to a solution of (R)-(+)-α-methylbenzyl)-dithiooxamide ((R)-α-MBAdto) (137.1 mg, 0.42 mmol) in 160 mL of 50% MeOH/CHCl_3_. An orange–yellow solution was obtained after stirring for 30 min; then, a sodium dithiolate solution of benzenecarbothioic acid, *S*,*S*′-1,2,5-thiadiazole-3,4-diyl ester (50.6 mg, 0.42 mmol) and sodium (21.2 mg, 0.92 mmol) was added drop-wise to the reaction mixture. After stirring the reaction mixture overnight, it was concentrated to 1/4 of the volume and filtered. A solution of Bu_4_NBr (135.4 mg, 0.42 mmol) in methanol was added to the filtrate. After slow evaporation of the filtrate, a solid formed, and it was collected by filtration (172.9 mg, 0.22 mmol, 53%). Analytical results are in accordance with the formula (Bu_4_N)[Ni((R)-α-MBAdt)(tdas)].

Elemental Analysis: calculated for C_36_H_55_N_5_NiS_5_ (776.89); C, 55.66; H, 7.14; N, 9.01; Ni, 7.55; S, 20.64; found: C, 55.38; H, 7.29; N, 9.25; Ni, 7.31; S, 20.29. UV-vis (CH_3_CN solution): λ (nm), ε (mol·cm^−1^·dm^−3^): 371, 7.9·10^3^; 563, 5.3 ·10^3^. FT–IR (ATR): ν_max_ (cm^−1^): 3146 (vw); 3060 (vw); 3031 (vw); 2955 (ms); 2927 (m); 2868 (w); 2801 (m); 1584 (m) 1516 (vs); 1484 (ms); 1446 (s); 1429 (s); 1379 (ms); 1344 (m); 1318 (vs); 1284 (m); 1233 (vs); 1172 (ms); 1152 (m); 1137 (m); 1110 (m); 1081 (m); 1025 (ms); 976 (vw); 904 (vw); 879 (m); 839 (vw); 800 (m); 786 (m); 770 (vs); 751 (ms); 738 (s); 695 (vs); 678 (ms); 548 (m); 496 (ms); 492 (vw); 486 (m); 416 (vw).

^1^H NMR (600 MHz, CD_3_CN) δ 7.42 (m, 4H), 7.37 (m, 4H), 7.29 (m, 2H), 5.23 (q, *J* = 6.8 Hz, 2H), 3.10–3.04 (m, 8H), 1.60 (m, 8H), 1.56 (d, *J* = 6.8 Hz, 6H), 1.39–1.31 (m, 8H), 0.97 (m, 12H).

#### 3.1.2. [Pd((R)-α-MBAdt)(DMSO)Cl]

A solution of Pd(DMSO)_2_Cl_2_ (250 mg, 0.75 mmol) in 20 mL methanol was added portion wise to a solution of N^1^,N^2^-bis((R)-1-phenylethyl)ethanebis(thioamide) (R)-α-MBAdto, (246 mg, 0.75 mmol) in 130 mL CHCl_3_. The obtained orange–yellow solution was then stirred overnight at room temperature. The orange–yellow powder of [Pd(R)-α-MBAdt)(DMSO)Cl] (392 mg, 96%) was collected by centrifugation. The product was used for the next step without further purification. FT–IR (KBr): ν_max_ (cm^−1^): 3193 (s); 3090 (s); 2980 (m); 2925 (m); 1568 (vs); 1529 (m); 1494 (w); 1447 (m); 1430 (vs); 1384 (s); 1347 (w); 1326 (w); 1214 (w); 1188 (m); 1087 (m); 1022 (w); 980 (vw); 915 (vw); 840 (vw); 760 (m); 698 (s), 540 (w).

#### 3.1.3. Bu_4_N[Pd((R)-α-MBAdt)(tdas)] (2)

A solution of Na (9.2 mg, 0.40 mmol) in 15 mL of methanol was added drop-wise to a stirred suspension of benzenecarbothioic acid, *S*,*S*′-1,2,5-thiadiazole-3,4-diyl ester (4.5 mg, 0.18 mmol) in 50 mL of 50% methanol/CHCl_3_. The mixture was stirred for 30 min at room temperature. The dithiolate solution was then added drop-wise to a yellow solution of [Pd((R)-α-MBAdt)(DMSO)Cl] (98.1 mg, 0.18 mmol) in 120 mL of 20% MeOH/CHCl_3_ at room temperature. The reaction mixture was stirred overnight, the obtained mixture was filtered, and the solvent was evaporated to 1/4 of the initial volume. Afterwards, a solution of Bu_4_NBr (58.0 mg, 0.18 mmol) in MeOH was added drop-wise, and the resulting mixture was roto-evaporated to dryness. The solid product was recrystallized in CH_3_CN/diethyl ether (80.1 mg, 0.097 mmol; yield 54%). Analytical results are in accordance with the formula (Bu_4_N)[Pd((R)-α-MBAdt)(tdas)].

Elemental Analysis: calculated for C_36_H_55_N_5_PdS_5_ (824.62); C, 52.44; H, 6.72; N, 8.49; Pd, 12.91; S, 19.44; found: C, 52.25; H, 6.62; N, 8.56; Pd, 12.99; S, 19.12. (CH_3_CN solution): λ (nm), ε (mol·cm^−1^·dm^−3^): 350, 14.5·10^3^; 448, 5.1·10^3^. FT–IR (ATR): ν_max_ (cm^−1^): 3177 (vw); 3060 (vw); 3031 (vw); 2955 (ms); 2927 (m); 2868 (m); 1592 (w) 1507 (s); 1449 (s); 1428 (ms); 1379 (ms); 1344 (m); 1312 (vs); 1229 (vs); 1167 (ms); 1136 (m); 1107 (ms); 1081 (m); 1022 (ms); 978 (vw); 904 (vw); 879 (m); 840 (vw); 791 (m); 776 (vs); 751 (ms); 738 (s); 695 (vs); 675 (ms); 620 (vw); 547 (m); 498 (ms); 482 (ms). ^1^H NMR (600 MHz, CD_3_CN) δ 7.43 (m, 4H), 7.38 (m, 4H), 7.30 (m, 2H), 5.30 (m, 2H), 3.11–3.04 (m, 8H), 1.60 (m, 14H), 1.38–1.31 (m, 8H), 0.97 (m, 12H).

#### 3.1.4. Bu_4_N[Pt((R)-α-MBAdt)(tdas)] (3)

Compound **3** was prepared following the same procedure employed for the synthesis of **2**, using 8.5 mg of Na (0.37 mmol in 10.0 mL of methanol), 60.9 mg of benzenecarbothioic acid, *S*,*S*′-1,2,5-thiadiazole-3,4-diyl ester (0.17 mmol in 50 mL of methanol/CHCl_3_), 107.8 mg of [Pt((R)-α-MBAdt)(DMSO)Cl] (0.16 mmol in 120 mL 20% MeOH/CHCl_3_), and 54.8 mg of Bu_4_NBr (0.31 mmol in MeOH). A total of 64.1 mg of product was obtained (0.070 mmol; yield 41.3%). Analytical results are in accordance with the formula (Bu_4_N)[Pt((R)-α-MBAdt)(tdas)].

Elemental Analysis: calculated for C_36_H_55_N_5_PtS_5_ (913.26); C, 47.35; H, 6.07; N, 7.67; Pt, 21.36; S, 17.56; found: C, 47.10; H, 6.25; N, 7.35; Pt, 21.51; S, 17.11. (CH_3_CN solution): λ (nm), ε (mol·cm^−1^·dm^−3^): 364, 10.3·10^3^; 505, 8.2·10^3^. FT–IR (KBr): ν_max_ (cm^−1^): 3194 (m); 2961 (ms); 2931 (m); 2872 (m); 1600 (vw); 1531 (m); 1503 (vs); 1468 (vs); 1447 (s); 1412 (m); 1377 (ms); 1344 (m); 1312 (vs); 1225 (vs); 1172 (s); 1090 (m); 1065 (ms); 1027 (ms); 1017 (ms); 978 (m); 913 (m); 884 (m); 846 (w); 790 (m); 772 (vs); 743 (vs); 732 (s); 699 (vs); 669 (m); 654 (ms); 607 (m); 547 (s); 491 (s); 405 (w). ^1^H NMR (600 MHz, CD_3_CN) δ 7.44 (m, 4H), 7.38 (m, 4H), 7.29 (m, 2H), 5.19 (q, *J* = 6.6 Hz, 2H), 3.03–3.06 (m, 8H), 1.69–1.50 (m, 14H), 1.40–1.30 (m, 8H), 0.96 (m, 12H).

### 3.2. Computational Studies

Density Functional Theory (DFT) [55] studies were performed using the GAUSSIAN 16 [56] software package. Throughout these investigations, the functional Becke three-parameter exchange and Lee–Yang–Parr correlation (B3LYP) [37,38] were employed, along with the basis set 6-31+G** [39] for H, C, N, and S atoms and the LANL2DZ [40] and Def2TZVPP [41,42] ECP basis sets for Ni and Pd and Pt, respectively. The geometry optimizations were carried out in both vacuum gas phase and acetonitrile solvent without any symmetry constraints. The effects of solvation were considered by the Polarizable Continuum Model (PCM) [57], whereas the absence of negative frequencies confirmed that the stationary points corresponded to minima on the potential energy surfaces. The 20 lowest singlet excited states of the investigated molecules in CH_3_CN were calculated within the time-dependent DFT (TD-DFT) formalism, as implemented in Gaussian [58,59]. The optimized geometries and the orbital isosurfaces (with isovalue plot 0.04) were visualized using ArgusLab 4.0.1 [60], whereas the simulate spectra and the orbital contributions to the electronic transitions were generated by GaussSum 3.0 [61].

### 3.3. Cellulose Film Sensor Preparation and Characterization

Cellulose film sensors were prepared by drop-casting a few drops of a solution of **1** (5 mg) dissolved in CH_2_Cl_2_ (6.4 g) on filter paper and drying in air. Diffuse reflectance UV–Vis–NIR characterization was performed with an Agilent Cary5000 spectrophotometer equipped with an external integrating sphere DRA2500.

### 3.4. Polymer Film: Preparation and Characterization

Composite thin films of **1**, **2**, and **3** in poly(methyl methacrylate) (PMMA; M̅_w_ ≈ 15,000, T_g_ = 86.5 °C, as determined by differential scanning calorimetry) were deposited using a Laurell Technologies WS-650_BT Series spin-coater, North Wales, United States, with the following spinning parameters: RPM1 = 700, ramp1 = 2 s, time1 = 5 s, RPM2 = 1000, ramp2 = 3 s, time2 = 21 s, on a glass slide substrate. Sample solutions were prepared in dichloromethane, keeping 5 wt % of the metal complexes with respect to PMMA, while the weight of PMMA was 10 wt % of the solvent.

Thin films of the HCl–adduct of **1**, **2**, and **3** in PMMA were obtained by exposing the films based on **1**, **2**, and **3** to HCl vapors (37%); the exposure time was of 30 min for Pd and Pt films and of 150 min for Ni films. On the other hand, the switching from the HCl–adduct to the original complex was achieved by exposing the HCl–adduct films to NH_3_ vapors (30%) with an exposure time of about 15–30 min.

Electronic absorption spectra of the thin films were recorded using a Shimadzu UV3600 spectrophotometer, while the thickness of the Ni, Pd, and Pt films was measured by an α-step stylus profilometer (Bruker Nano, Inc. DektakXT, Santa Barbara, CA, USA), resulting in 1890 ± 49, 1670 ± 43, and 2180 ± 57 nm, respectively.

### 3.5. Corona-Wire Poling Setup

The fundamental incident light was generated by a 1064 nm Q-switched Nd:YAG (Quanta System S.p.A. Giant G790-20, Samarate, Italy) laser with a pulse of 7 ns and a 20 Hz repetition rate. The output pulse was attenuated to 0.50 mJ and focused with a lens (f = 600 mm) on the sample, placed over the hot stage. The corona poling process was performed inside a drybox under a N_2_ atmosphere. The fundamental beam was polarized in the incidence plane (so-called p-polarized) with an angle of about 55° with respect to the sample in order to optimize the second-harmonic generation (SHG) signal. The hot-stage temperature was controlled by a GEFRAN 800 controller, while the corona-wire voltage (up to 10 kV across a 10 mm gap) was applied by a TREK 610E high-voltage supply. After rejection of the fundamental beam by an interference filter and a glass cutoff filter, the p-polarized SHG signal at 532 nm was detected with a UV−vis photomultiplier (PT, Hamamatsu Photonics K.K. C3830, Hamamatsu, Shizuoka, Japan). The output signal from the PT was set to a digital storage oscilloscope and then processed by a computer with dedicated software.

### 3.6. Maker Fringe Measurement

In the Maker fringe experiment, the second-harmonic intensity was detected as a function of the incidence angle θ of the fundamental beam and normalized with respect to that of a 1 mm thick calibrated quartz crystal wafer (X-cut), whose d_11_ value was 0.46 pm V^−1^. In order to determine the nonzero independent components of the susceptibility tensor (χ_ij_^(2)^) for the poled films (C_∞v_ symmetry), Maker fringe measurements were conducted with different polarizations of the fundamental and second-harmonic beam: p → p, s → p, and 45° → s (where p and s indicate the polarization of the beam in the plane parallel and orthogonal to the incident beam, respectively) [47,62].

## 4. Conclusions

In heteroleptic d^8^-metal dithiolenes, the tunability of the donor (dithiolato) and acceptor (dithiooxamide and dithiooxamidate) components, exhibiting different electronic and/or structural features varying from rigid and planar systems to conformationally flexible ones, allows for tuning the linear and nonlinear optical properties in these chromophores. In the case of the Bu_4_N[M((*R*)-α-MBAdt)(tdas)] [M = Ni (**1**), Pd (**2**), Pt (**3**)] triad, described here, it is shown that the tdas donor ligand allows for rationally extending the color palette of their acido-chromic response and for promoting the nano-organization of these kinds of NLO-phores in polymeric matrixes. Remarkably, the obtained multicolor HX–receptor complexes were deposited on a cellulose substrate and work as sensors for the naked-eye detection of hydrohalic acids or, in the tight ion-paired adduct form, of visible transparent amines both in solution and on paper substrates. Moreover, films characterized by a reversible chemically switchable stable and large second-harmonic generation were obtained.

The reported achievements can stimulate future work to exploit the potential of this class of complexes on the variation in the D and A components as linear and nonlinear optical molecular sensors and switches and to optimize the processing procedures to achieve good SHG responses in bulk.

In summary, the successful preparation of a Ni complex, the processing of the pure complexes as films on paper or dispersed in PMMA on glass substrates capable of detecting volatile hydrohalic acids and organic amines with naked-eye color changes, and the ability of **1–3** to be doped into polymer matrixes forming films able to reversibly and repeatably respond to external stimuli seem like promising results for providing versatile candidates for smart optoelectronic devices and chemo-sensors.

## Figures and Tables

**Table 1:** Absorption maxima of Bu_4_N[M((*R*)-α-MBAdt)(L)] derivatives upon addition of HCl.

Complex	λ_max_ (nm) ^a^ [ε] ^b^	λ_max_ (nm) ^c^ [ε] ^b^	
Bu_4_N[Pd((*R*)-α-MBAdt)(tdas)] (**2**)	454 [4.9]	601 [4.9]	This work
Bu_4_N[Pt((*R*)-α-MBAdt)(tdas)] (**3**)	513 [7.6]	651 [10.9]	This work
Bu_4_N[Pd((*R*)-α-MBAdt)(dmit)] (**4**)	525 [15.7]	747 [6.7]	Ref. [21]
Bu_4_N[Pt((*R*)-α-MBAdt)(dmit)] (**5**)	543 [9.6]	781 [9.6]	Ref. [21]
Bu_4_N[Pd((*R*)-α-MBAdt)(ddmedt)] (**6**)	525 [4.0]	715 [5.7]	Ref. [21]
Bu_4_N[Pt((*R*)-α-MBAdt)(ddmedt)] (**7**)	558 [5.5]	764 [11.1]	Ref. [21]
Bu_4_N[Pd((*R*)-α-MBAdt)(quinoxdt)] (**8**)	550 [5.5]	773 [5.8]	Ref. [21]
Bu_4_N[Pt((*R*)-α-MBAdt)(quinoxdt)] (**9**)	606 [7.8] ^d^	814 [12.4]	Ref. [21]

[a] DMF; [b] ε × 10^3^ (M^−1^ cm^−1^); [c] DMF + HCl; [d] in ACN, λ_max_~MF.

**Table 2:** Coefficients of the second-order susceptibility tensor χ_ij_^(2)^ for Ni (**1**), Pd (**2**), and Pt (**3**) films and related HCl–adduct films with their respective chemical SHG switch.

Sample	χ_33_ (pm/V)	χ_31_ (pm/V)	χ_15_ (pm/V)
**1** film	1.8	0.64	0.41
**1** film + HCl	2.78	0.84	0.66
HCl–adduct **1** film	3.20	0.66	0.55
HCl–adduct **1** film + NH_3_	2.08	0.34	0.16
**2** film	3.24	0.78	0.32
**2** film + HCl	5.94	1.26	0.74
HCl–adduct **2** film	4.22	1.04	0.72
HCl–adduct **2** film + NH_3_	2.82	0.82	0.42
**3** film	6.12	1.28	0.69
**3** film + HCl	8.06	2.06	1.02
HCl–adduct **3** film	8.48	2.48	2.02
HCl–adduct **3** film + NH_3_	6.40	1.92	1.14

## Data Availability

The original contributions presented in the study are included in the article and Supplementary Materials; further inquiries can be directed to the corresponding authors.

## Supplementary Materials

The following supporting information can be downloaded at: https://www.mdpi.com/article/10.3390/molecules30194004/s1, Figure S1: FT-IR spectra; Figures S2–S4: 1H-NMR spectra; Figure S5: Electronic spectra; Figure S6: DFT-optimized molecular structures; Tables S1–S3: DFT-calculated molecular orbitals; Figures S7–S9: Calculated absorption spectra; Tables S4–S6: TD-DFT-calculated energy transitions; Figure S10: Diffuse reflectance spectra; Figure S11: Photographs of 1 drop-casted on glass support; Figure S12: Spectral response of the paper sensor based on **1**; Figure S13: UV−vis absorption spectra of **1** and **3** films; Figure S14: Corona-wire poling dynamics of **1** and **3** films.
