# Supplementary material for: HX-Linear and Nonlinear Optical Responsiveness of Rationally Designed Heteroleptic d8-Metallo-dithiolene Complexes
Source: Molecules. 2025 Oct 7;30(19):4004. doi: 10.3390/molecules30194004 (PMC12526242; doi:10.3390/molecules30194004)
Supplement: Supplementary file 1 [file molecules-30-04004-s001.zip › molecules-3897888-supplementary.pdf]

Supplementary Material

for

HX-linear and nonlinear optical responsiveness of rationally designed heteroleptic d^8-metallo-dithiolene complexes

Salahuddin S. Attar,[a,#] Flavia Artizzu,[b] Luca Pilia,[c,*] Angela Serpe,[d] Alessia Colombo,[e,*] Claudia Dragonetti,[e,f] Francesco Fagnani,[e] Dominique Roberto,[e,f] Daniele Marinotto[f,g] and Paola Deplano[a]

[a] Dipartimento di Scienze Chimiche e Geologiche, Università di Cagliari, 09042 Monserrato (CA) (Italy)

[b] Department of Sustainable Development and Ecological Transition (DISSTE), University of Eastern Piedmont, Vercelli, Italy

[c] Dipartimento di Ingegneria Meccanica, Chimica e dei Materiali, Università di Cagliari, Via Marengo 2, 09123 Cagliari (Italy).

[d] Dipartimento di Ingegneria Civile, Ambientale e Architettura, Università di Cagliari, Via Marengo 2, 09123 Cagliari (Italy)

[e] Dipartimento di Chimica, Università degli Studi di Milano e UdR-INSTM di Milano, Via C. Golgi 19, I-20133 Milan, Italy

[f] CIMaINa, Università degli Studi di Milano, via G. Celoria 16, I-20133 Milan, Italy

[g] Istituto di Scienze e Tecnologie Chimiche "Giulio Natta" (SCITEC), Consiglio Nazionale delle Ricerche (CNR), via C. Golgi 19, 20133 Milan, Italy

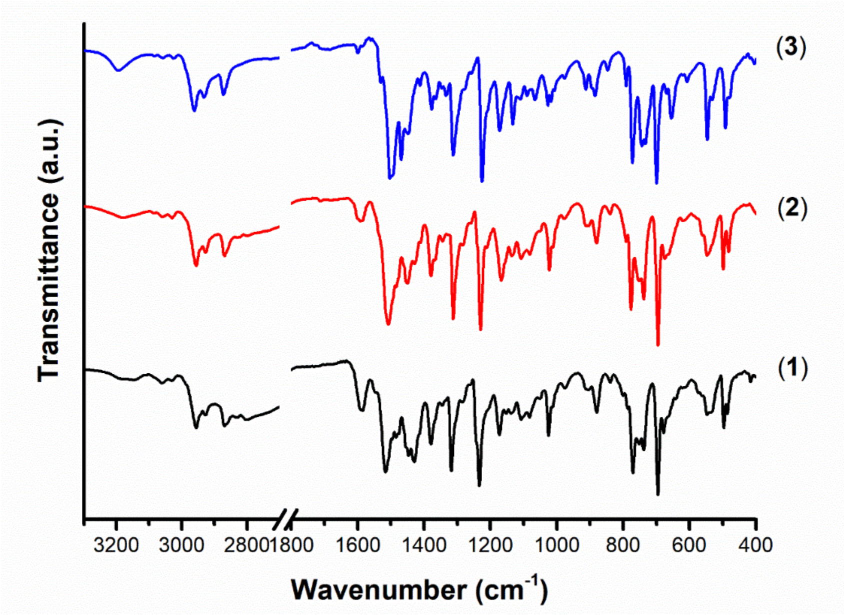

Figure S1. FT-IR spectra of **1**, **2** and **3** on measured by ATR method.

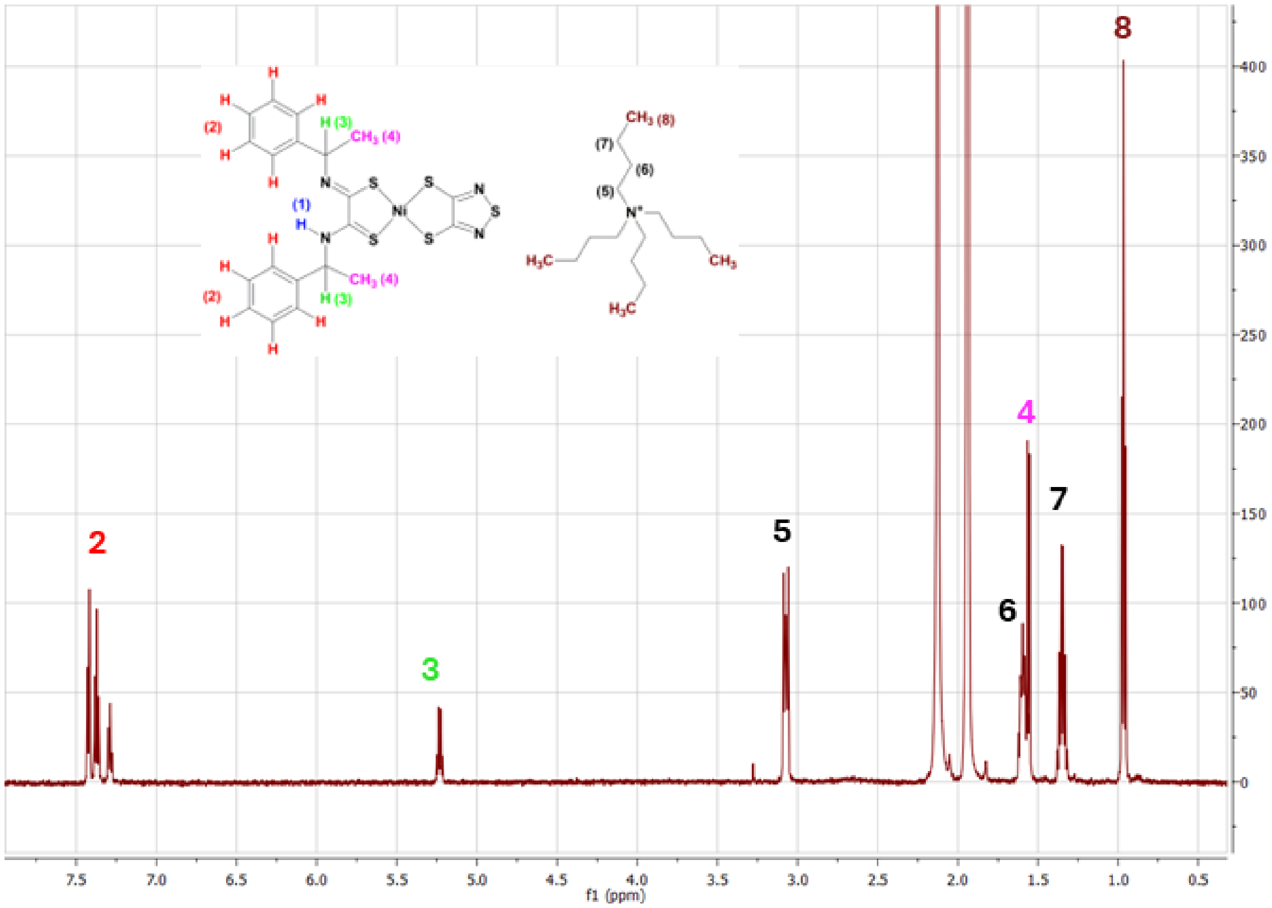

Figure S2. ^{1}H-NMR spectrum of $Bu_4N[Ni((R)\text{-}\alpha\text{-}MBAdt)(tdas)]$ in CD_3CN solution. In the spectrum the signal related to proton (1) is not detectable due to rapid exchange.

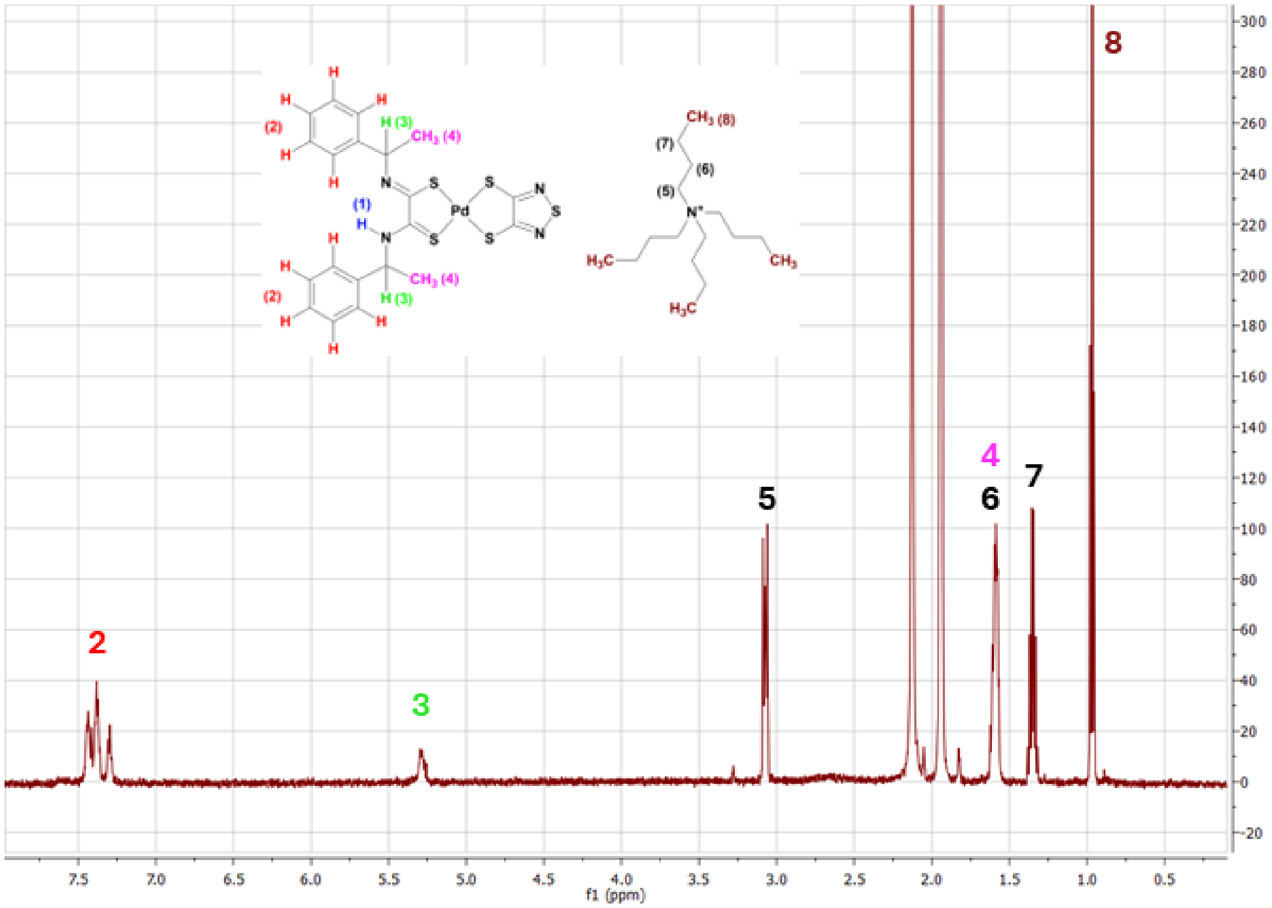

Figure S3. ^{1}H-NMR spectrum of $Bu_4N[Pd((R)\text{-}\alpha\text{-}MBAdt)(tdas)]$ in CD_3CN solution. In the spectrum the signal related to proton (1) is not detectable due to rapid exchange.

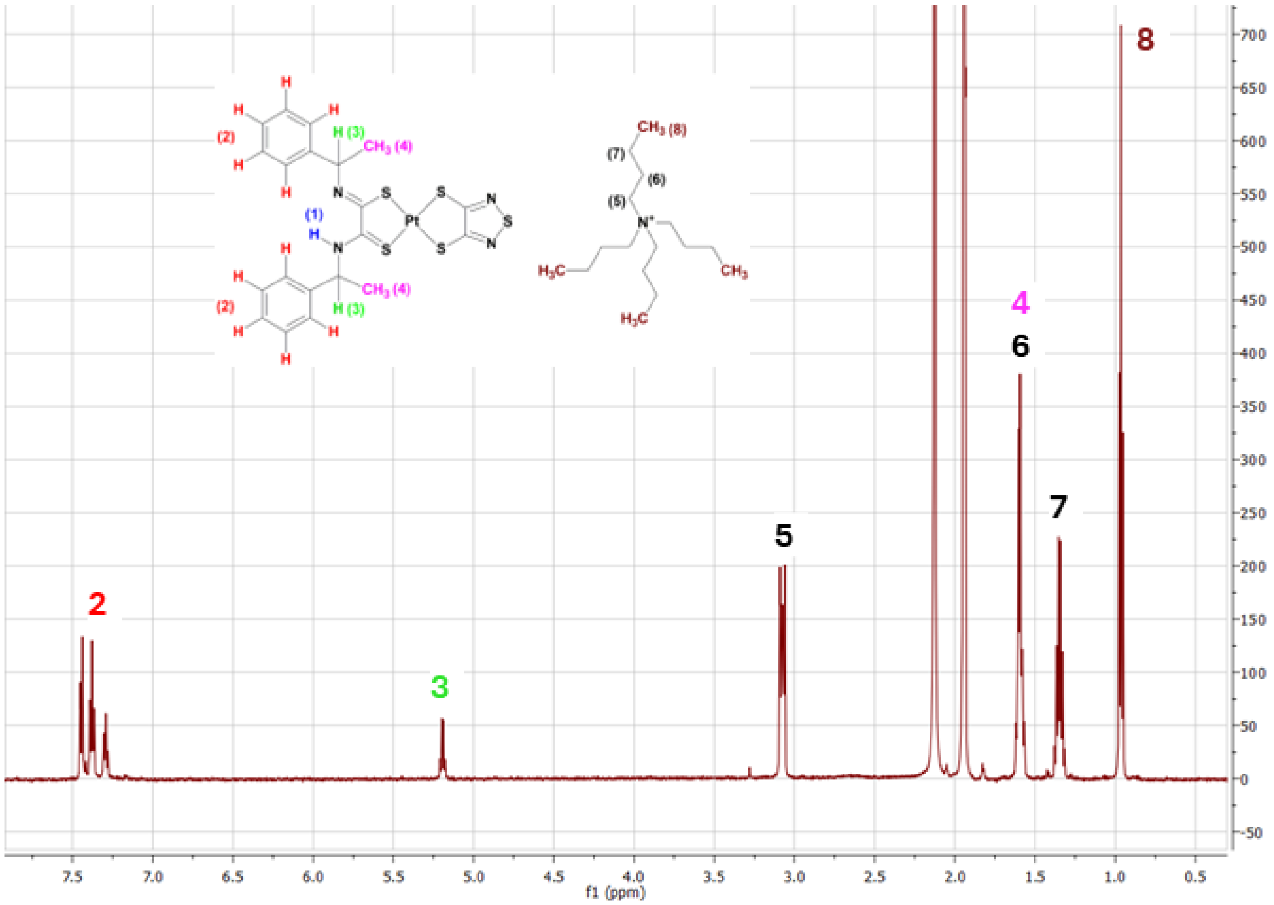

Figure S4. ^{1}H-NMR spectrum of $Bu_4N[Pt((R)\text{-}\alpha\text{-}MBAdt)(tdas)]$ in CD_3CN solution. In the spectrum the signal related to proton (1) is not detectable due to rapid exchange.

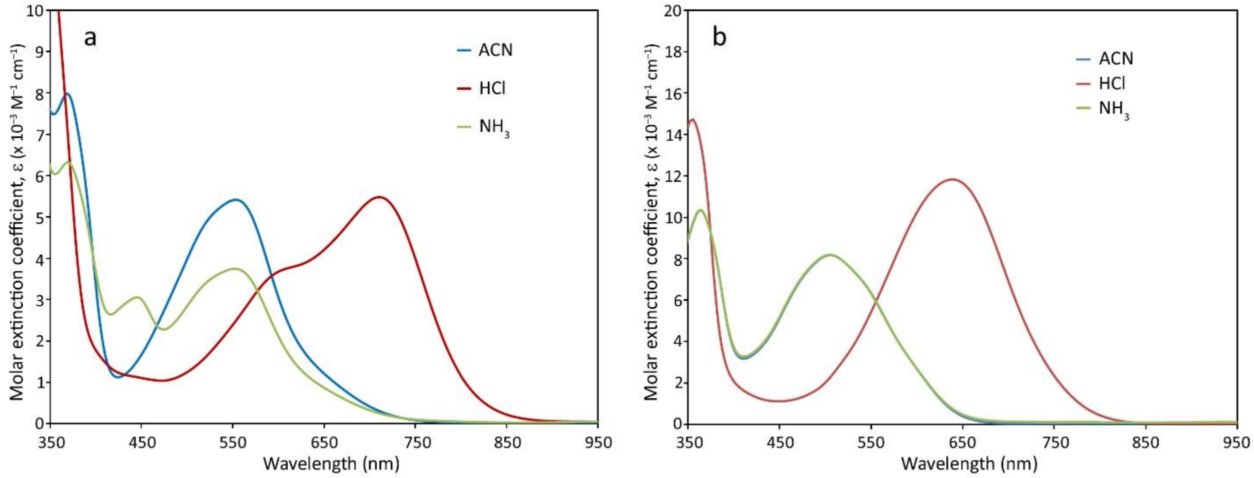

Figure S5. Electronic absorption spectra of a) **1** and b) **3**, in ACN (blue) upon the sequential addition of an equimolar amount of HCl (red) and NH_3 (blue).

Computational studies

Density Functional Theory (DFT) [1] studies were performed by GAUSSIAN 16 [2] software package. Throughout these investigations the functional Becke three-parameter exchange and Lee–Yang–Parr correlation (B3LYP) [3, 4] have been employed, along with the basis set 6-31+G** [5] for H, C, N and S atoms, and the LANL2DZ [6] and Def2TZVPP [7, 8] ECP basis sets for Ni and Pd and Pt, respectively. The geometry optimizations were done in both vacuum gas phase and acetonitrile solvent, without any symmetry constraints. The effects of solvation were considered by the Polarizable Continuum Model (PCM) [9], whereas the absence of negative frequencies confirmed that the stationary points correspond to minima on the potential energy surfaces. The 20 lowest singlet excited states of the investigated molecules in CH_3CN were calculated within the time-dependent DFT (TD-DFT) formalism as implemented in Gaussian [10, 11]. The optimized geometries and the orbital isosurfaces (with isovalue plot 0.04) were visualized using ArgusLab 4.0.1 [12], whereas the simulate spectra and the orbitals contributions to the electronic transitions were generated by GaussSum 3.0 [13].

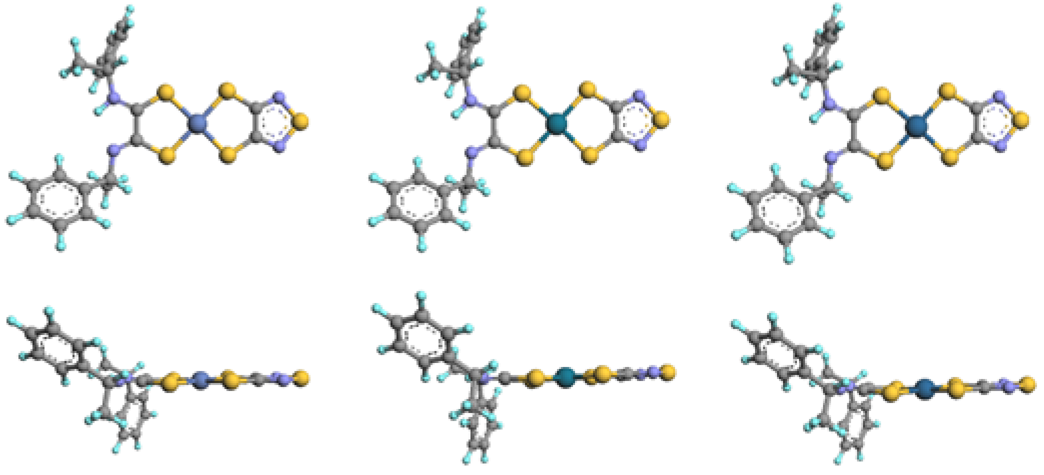

(1) **(2)** **(3)**

Figure S6. DFT optimized molecular structures of complexes **1**, **2** and **3** in CH_3CN solution.

Table S1. DFT calculated molecular orbitals of $[Ni((\mathit{R})\text{-}\alpha\text{-MBAdt})(tdas)]^{-1}$ in CH_3CN (isovalue plot 0.04).

LUMO+5 -0.49	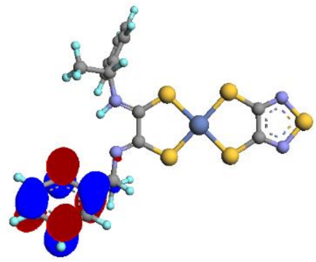	LUMO+4 -0.55	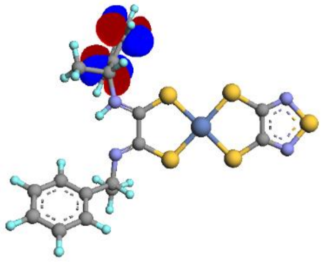
LUMO+3 -0.62	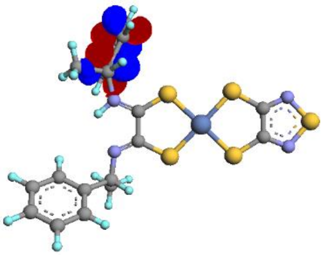	LUMO+2 -1.28	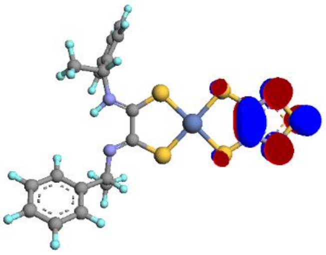
LUMO+1 -2.21	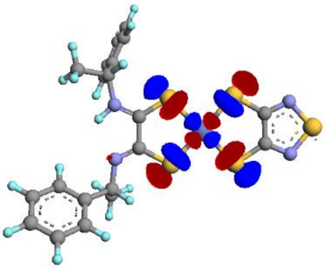	LUMO -2.41	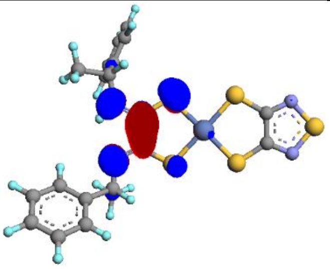
HOMO -5.41	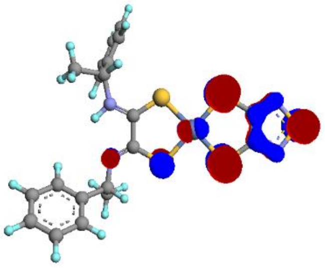	HOMO-1 -5.92	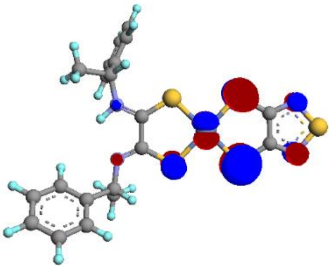
HOMO-2 -6.23	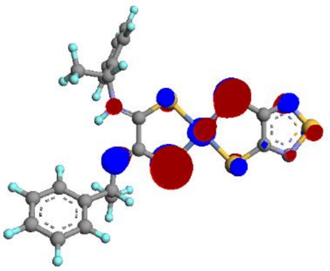	HOMO-3 -6.25	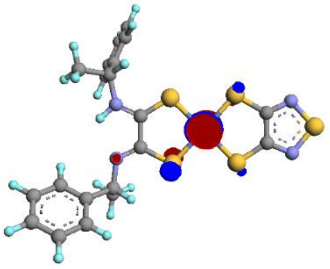
HOMO-4 -6.75	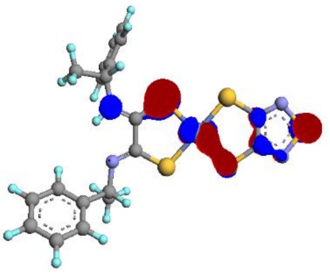	HOMO-5 -6.80	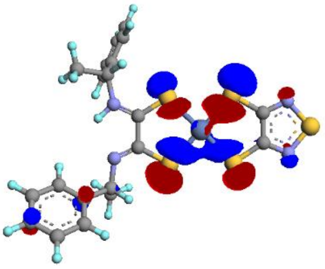

Table S2. DFT calculated molecular orbitals of [Pd((*R*)-α-MBAdt)(tdas)]$^{-1}$ in CH_3CN (isovalue plot 0.04).

LUMO+5<br>-0.49	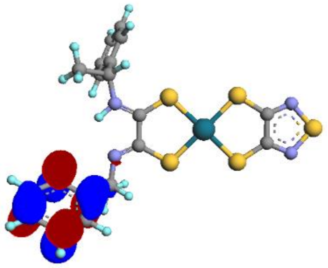	LUMO+4<br>-0.56	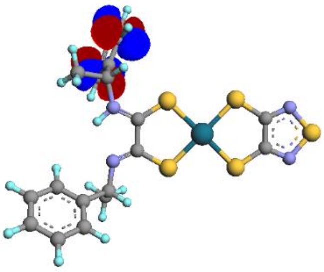
LUMO+3<br>-0.62	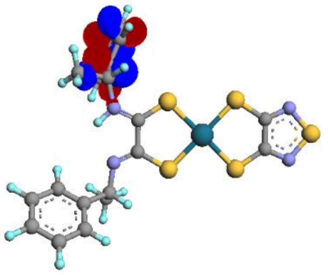	LUMO+2<br>-1.31	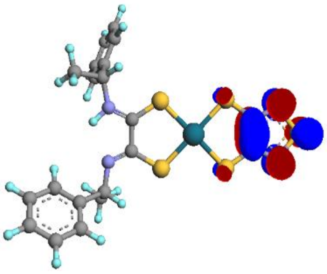
LUMO+1<br>-2.05	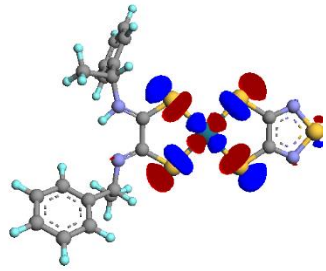	LUMO<br>-2.41	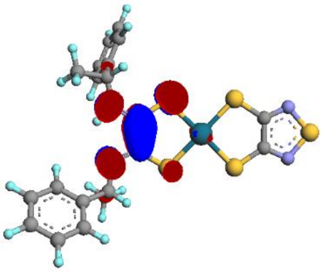
HOMO<br>-5.35	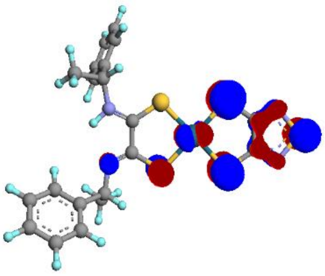	HOMO-1<br>-5.84	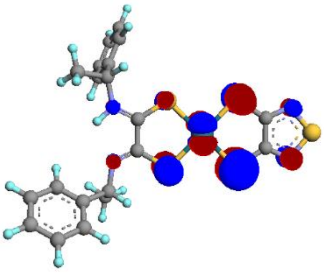
HOMO-2<br>-6.31	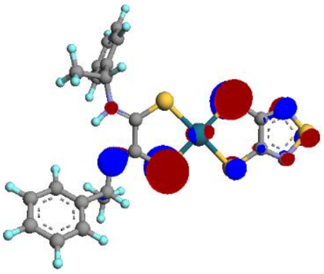	HOMO-3<br>-6.46	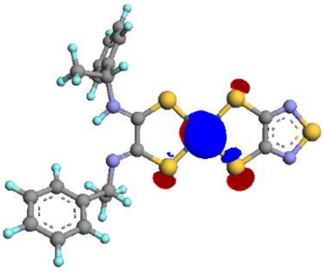
HOMO-4<br>-6.76	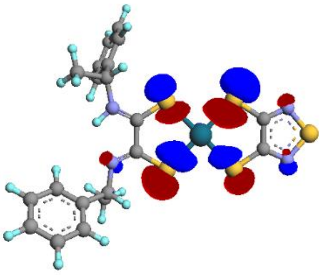	HOMO-5<br>-6.85	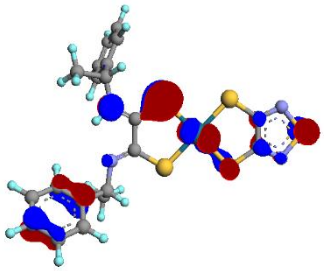

Table S3. DFT calculated molecular orbitals of [Pt((*R*)-α-MBAdt)(tdas)]$^{-1}$ in CH_3CN (isovalue plot 0.04).

LUMO+5 -0.49	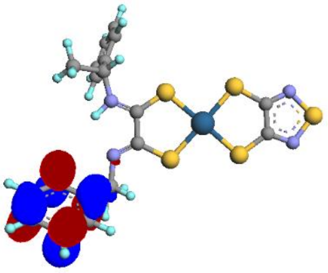	LUMO+4 -0.55	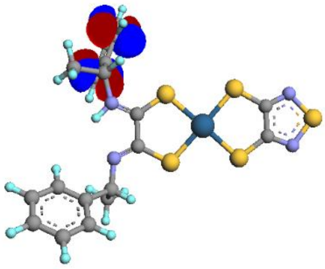
LUMO+3 -0.61	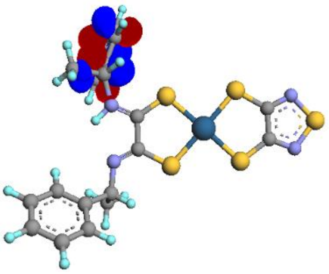	LUMO+2 -1.34	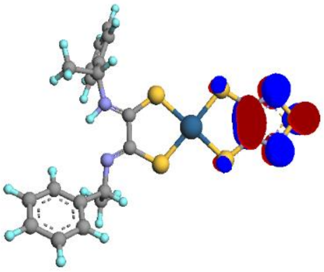
LUMO+1 -1.38	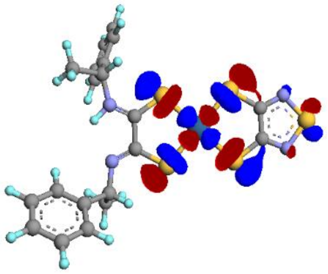	LUMO -2.42	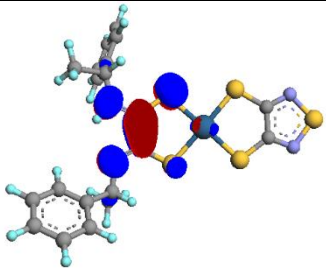
**HOMO** **-5.17**	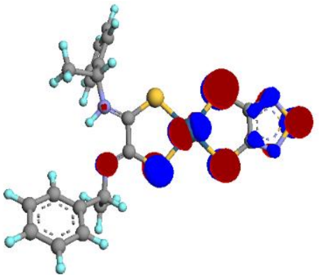	HOMO-1 -5.57	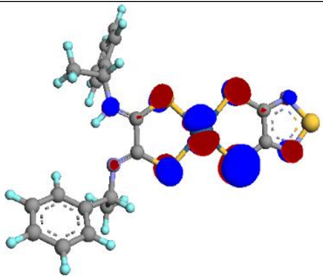
HOMO-2 -6.32	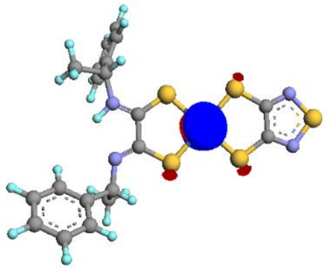	HOMO-3 -6.35	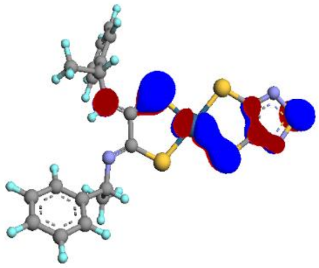
HOMO-4 -6.76	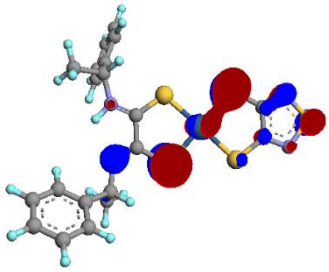	HOMO-5 -6.85	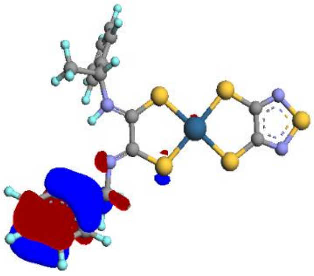

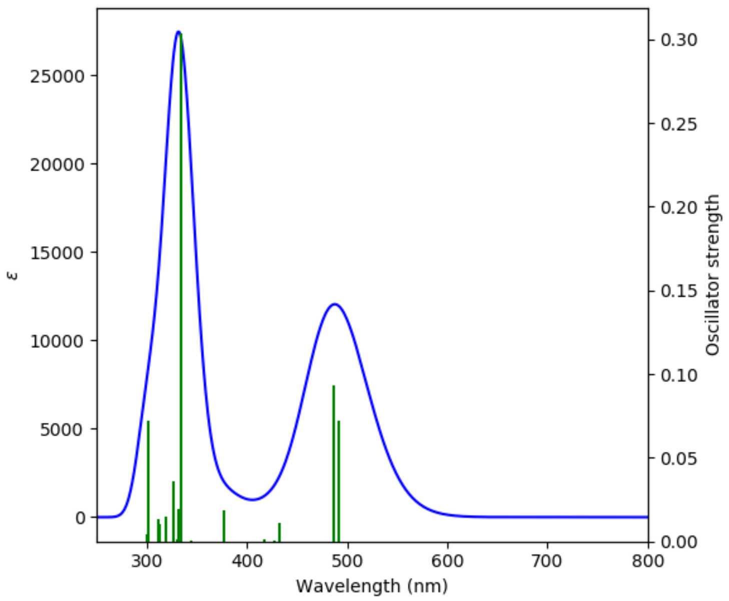

Figure S7. Calculated absorption spectrum for **1** in acetonitrile solution.

Table S4. TD-DFT calculated lowest energy transitions for **1** in CH_3CN solution.

Exp. absorption (nm)	Calc. absorption (nm)	f^a	Major contributions
563	491	0.0722	H-19->L+1 (11%), H-13->L+1 (20%), H-11->L+1 (15%), HOMO->LUMO (42%)
	486	0.0932	H-19->L+1 (11%), H-13->L+1 (19%), H-11->L+1 (10%), HOMO->LUMO (52%)
	432	0.0108	H-1->LUMO (94%) HOMO->LUMO (2%)
371	377	0.0188	H-3->LUMO (10%), H-2->LUMO (81%)
	334	0.3038	H-4->LUMO (36%), HOMO->L+2 (53%)
	331	0.0192	H-12->L+1 (24%), H-11->LUMO (32%), H-6->LUMO (10%)
	327	0.0361	H-4->LUMO (45%), HOMO->L+2 (36%)
	319	0.0145	H-7->LUMO (35%), H-6->LUMO (51%)
	313	0.0103	H-11->LUMO (14%), H-8->LUMO (12%), H-7->LUMO (43%), H-6->LUMO (20%)
	312	0.0136	H-1->L+2 (80%) H-10->L+1 (6%)
	302	0.0724	H-10->LUMO (28%), H-9->LUMO (45%), H-8->LUMO (11%)

[a] Calculated oscillator strength

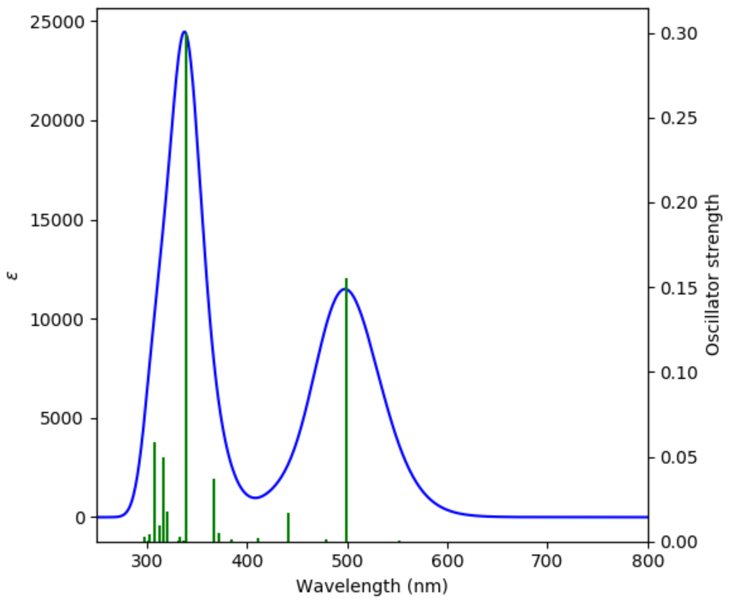

Figure S8. Calculated absorption spectrum for **2** in acetonitrile solution.

Table S5. TD-DFT calculated lowest energy transitions for **2** in CH_3CN solution.

Exp. absorption (nm)	Calc. absorption (nm)	f^a	Major contributions
448	499	0.1550	HOMO->LUMO (93%)
	441	0.0166	H-3->L+1 (12%), H-1->LUMO (82%) HOMO->LUMO (3%)
350	366	0.0369	H-2->LUMO (73%), H-2->L+1 (21%)
	339	0.2999	HOMO->L+2 (90%)
	320	0.0177	H-7->LUMO (35%), H-6->LUMO (37%), H-5->LUMO (16%) H-10->LUMO (7%)
	308	0.0587	H-6->LUMO (15%), H-5->LUMO (62%) H-1->L+2 (7%)

[a] Calculated oscillator strength

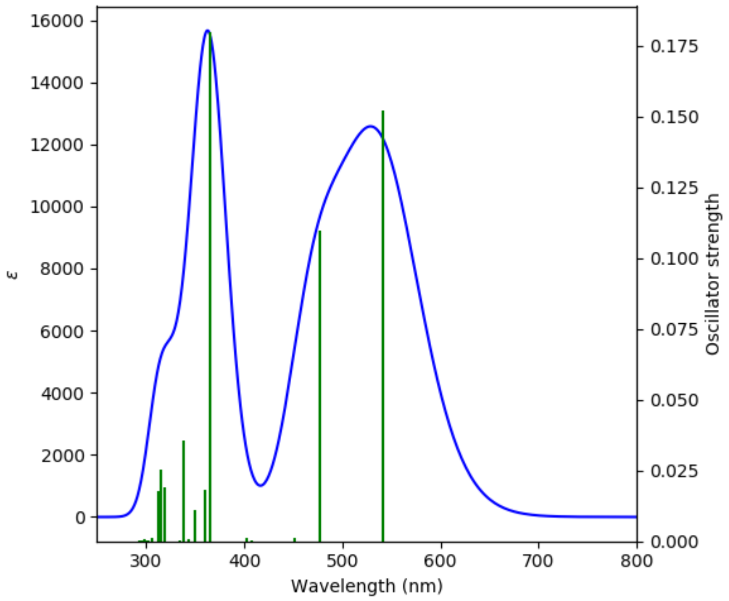

Figure S9. Calculated absorption spectrum for **3** in acetonitrile solution.

Table S6. TD-DFT calculated lowest energy transitions for **3** in CH_3CN solution.

Exp. absorption (nm)	Calc. absorption (nm)	f^a	Major contributions
505	541	0.152	H-1->LUMO (12%), HOMO->LUMO (85%)
	477	0.1095	H-1->LUMO (86%), HOMO->LUMO (12%)
364	365	0.1799	H-3->LUMO (33%), HOMO->L+2 (61%)
	360	0.0181	H-3->LUMO (57%), HOMO->L+2 (27%) H-1->L+2 (5%)
	350	0.0111	H-2->L+1 (82%), H-1->L+2 (5%)
	339	0.0355	H-1->L+2 (85%) H-2->L+1 (7%)
	319	0.0191	H-7->LUMO (27%), H-5->LUMO (62%)
	315	0.0251	H-4->LUMO (83%)
	313	0.0179	H-9->LUMO (16%), H-8->LUMO (37%), H-7->LUMO (32%) H-6->LUMO (5%), H-5->LUMO (6%)

[a] Calculated oscillator strength

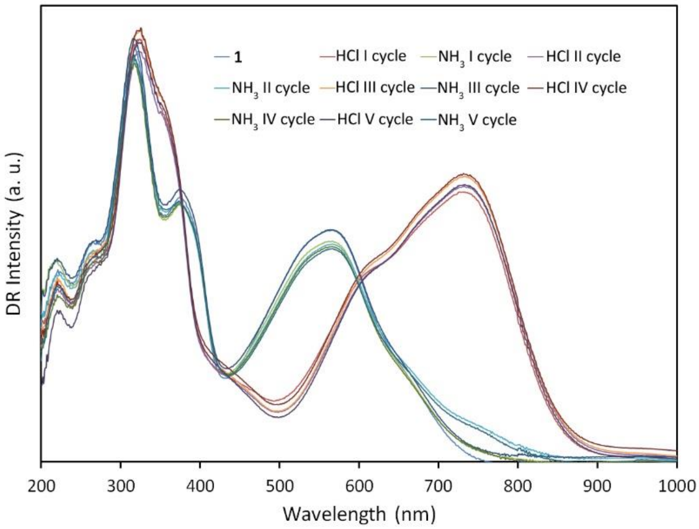

Figure S10. Diffuse reflectance (DR) spectra of **1** on cellulose support upon five cycles of sequential exposure to HCl and NH_3 vapors.

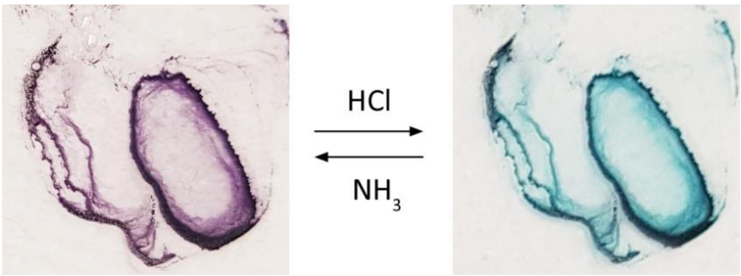

Figure S11. Photographs of **1** directly dropcasted on glass support and exposed to HCl and NH_3 vapors.

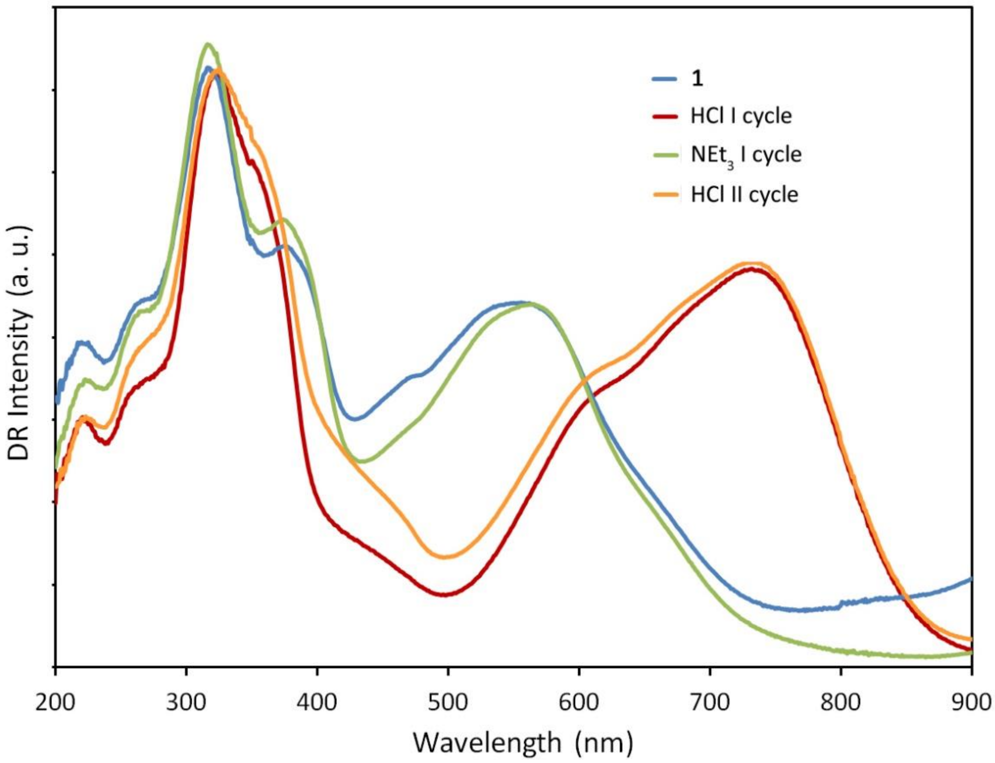

Figure S12. Spectral response of the paper sensor based on **1** sequentially exposed to HCl and triethylamine (NEt_3) vapors.

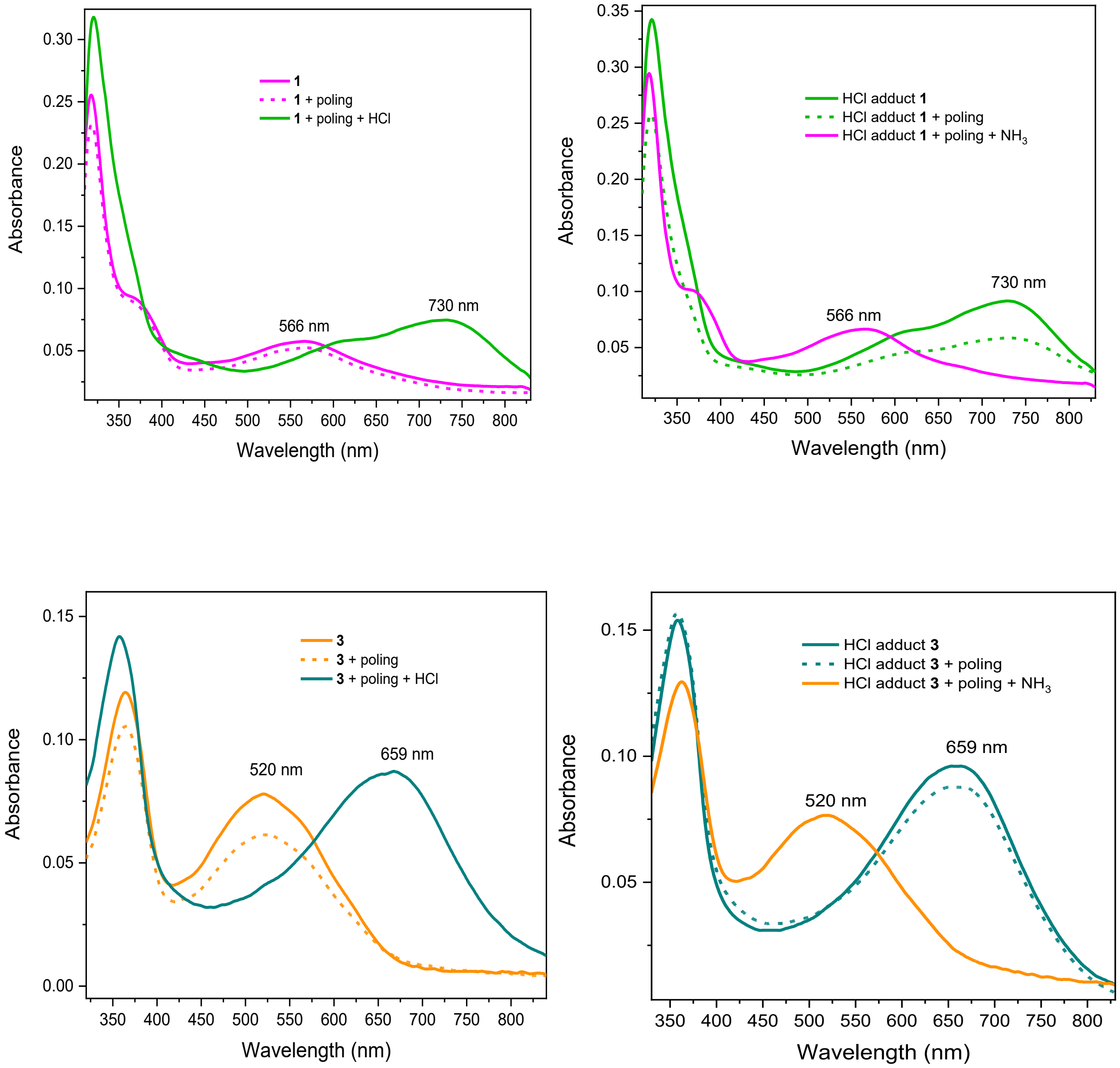

Figure S13. UV−vis absorption spectra of Ni (**1**) and Pt (**3**) films (left) and related HCl-adducts films (right) before and after poling with relative switch.

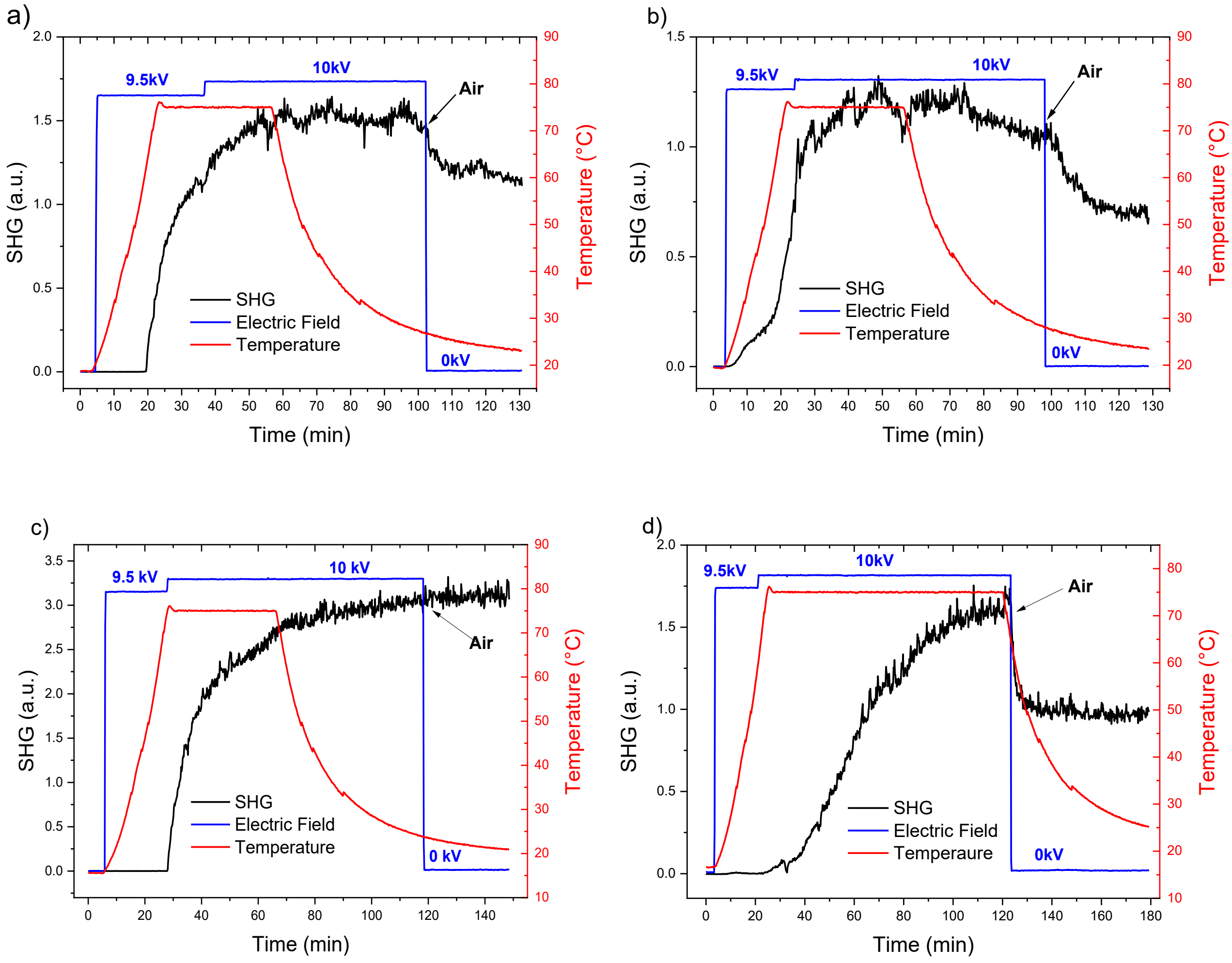

Figure S14. Corona-wire poling dynamics of (a) **1** film, (b) HCl-adduct **1** film, (c) **3** film, (d) HCl-adduct **3** film.

ReferencesR.G. Parr, W. Yang, *Density Functional Theory of Atoms and Molecules*, Oxford University Press: Oxford, UK, **1989**.

2. M.J. Frisch, G.W. Trucks, H.B. Schlegel, G.E. Scuseria, M.A. Robb, J.R. Cheeseman, G. Scalmani, V. Barone, G.A. Petersson, H. Nakatsuji, et al. Gaussian 16, Revision C.01, Gaussian, Inc.: Wallingford, CT, USA, **2016**.
3. D. Becke, *Density-functional thermochemistry. III. The role of exact exchange*. J. Chem. Phys. **1993**, 98, 5648–5652. DOI: 10.1063/1.464913.
4. C. Lee, W. Yang, R.G. Parr, *Development of the Colle-Salvetti correlation-energy formula into a functional of the electron density*. Phys. Rev. B **1988**, 37, 785–789. DOI: 10.1103/physrevb.37.785.
5. P.C. Hariharan, J.A. Pople, *The influence of polarization functions on molecular orbital hydrogenation energies*. Theoret. Chim. Acta **1973**, 28, 213–222. DOI: 10.1007/BF00533485.
6. P.J. Hay, W.R. Wadt, *Ab initio effective core potentials for molecular calculations. Potentials for K to Au including the outermost core orbitals*. J. Chem. Phys. **1985**, 82, 299–310. DOI: 10.1063/1.448975.

7. F. Weigend, R. Ahlrichs, *Balanced basis sets of split valence, triple zeta valence and quadruple zeta valence quality for H to Rn: Design and assessment of accuracy.* Phys. Chem. Chem. Phys. **2005,** 7, 3297-305. DOI: 10.1039/B508541A.
8. F. Weigend, *Accurate Coulomb-fitting basis sets for H to Rn*. Phys. Chem. Chem. Phys. **2006,** 8, 1057-65. DOI: 10.1039/B515623H.
9. S. Miertuš, E. Scrocco, J. Tomasi, *Electrostatic Interaction of a Solute with a Continuum. A Direct Utilization of ab initio Molecular Potentials for the Prevision of Solvent Effects.* Chem. Phys. **1981**, 55, 117-29. DOI: 10.1016/0301-0104(81)85090-2.
10. G. Scalmani, M. J. Frisch, B. Mennucci, J. Tomasi, R. Cammi, V. Barone, *Geometries and properties of excited states in the gas phase and in solution: Theory and application of a time-dependent density functional theory polarizable continuum model.* J. Chem. Phys. **2006**, 124, 094107. DOI: 10.1063/1.2173258.
11. M. Cossi, N. Rega, G. Scalmani, V. Barone, *Energies, structures, and electronic properties of molecules in solution with the C-PCM solvation model.* J. Comp. Chem. **2003**, 24, 669. DOI: 10.1002/jcc.10189.
12. M.A. Thompson, ArgusLab 4.0.1, Planaria Software LLC: Seattle, WA, USA, **2021**. Available online: http://www.arguslab.com/arguslab.com/ArgusLab.html/.
13. N.M. O'Boyle, A.L. Tenderholt, K.M. Langner, *cclib: A library for package-independent computational chemistry algorithms.* J. Comp. Chem. **2008**, 29, 839-845. DOI: 10.1002/jcc.20823.
